# Allosteric modulation of the farnesoid X receptor by a small molecule

**DOI:** 10.1038/s41598-018-25158-5

**Published:** 2018-05-01

**Authors:** Matthias Gabler, Jan Kramer, Jurema Schmidt, Julius Pollinger, Julia Weber, Astrid Kaiser, Frank Löhr, Ewgenij Proschak, Manfred Schubert-Zsilavecz, Daniel Merk

**Affiliations:** 1 Institute of Pharmaceutical Chemistry, Goethe-University Frankfurt, Max-von-Laue-Str. 9, D-60438 Frankfurt, Germany; 2 Institute of Biophysical Chemistry, Goethe-University Frankfurt, Max-von-Laue-Straße 9, D-60438 Frankfurt, Germany

## Abstract

The bile acid activated transcription factor farnesoid X receptor (FXR) regulates numerous metabolic processes and is a rising target for the treatment of hepatic and metabolic disorders. FXR agonists have revealed efficacy in treating non-alcoholic steatohepatitis (NASH), diabetes and dyslipidemia. Here we characterize imatinib as first-in-class allosteric FXR modulator and report the development of an optimized descendant that markedly promotes agonist induced FXR activation in a reporter gene assay and FXR target gene expression in HepG2 cells. Differential effects of imatinib on agonist-induced bile salt export protein and small heterodimer partner expression suggest that allosteric FXR modulation could open a new avenue to gene-selective FXR modulators.

## Introduction

**Figure 1:**
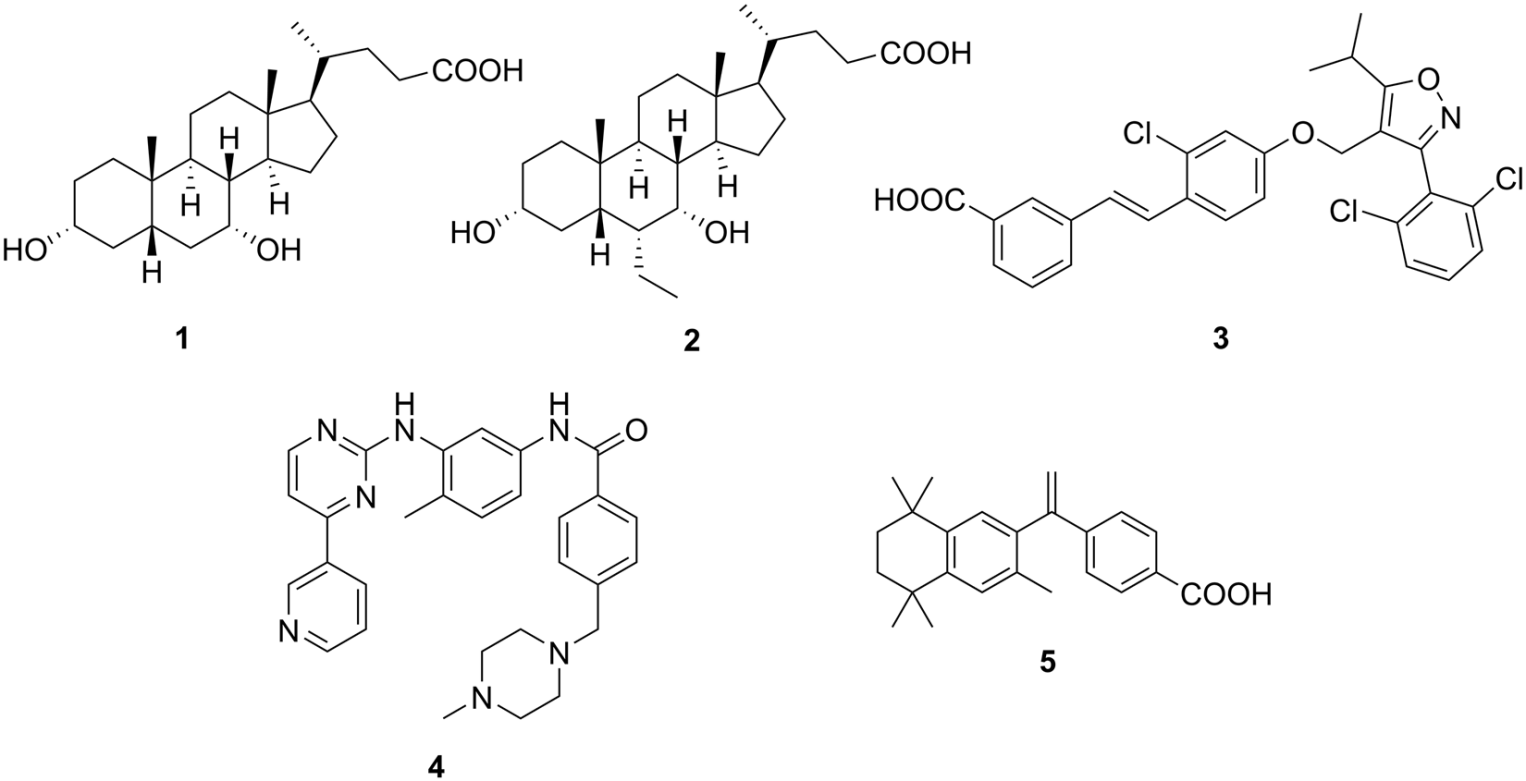
Chemical structures of endogenous FXR agonist chenodeoxycholic acid (**1**, CDCA), first-in-class approved FXR targeting agent 6α-ethyl-CDCA (**2**, 6-ECDCA, INT-747, obeticholic acid, OCA) the widely-used reference FXR agonist GW4064 (**3**), imatinib (**4**, CGP 57148B, STI 571, Gleevec^®^) and retinoid X receptor agonist bexarotene (**5**).

Farnesoid X receptor (FXR) is a member of the nuclear receptor superfamily predominantly found in liver, intestine and kidney^1–4^. As ligand-activated transcription factor, FXR controls metabolic and inflammatory pathways as monomer or as a permissive heterodimer with the retinoid X receptor (RXR)^1–4^. Bile acids, such as chenodeoxycholic acid (**1**, CDCA, Fig. 1) act as endogenous ligands of FXR thereby regulating their own biosynthesis, transport and metabolism in a negative feedback loop^5^. For its involvement in metabolism, liver health and inflammation, FXR has considerable pharmacological relevance^5–7^. Many beneficial effects of FXR activation are mediated by up-regulation of the nuclear receptor small heterodimer partner (SHP) which lacks a DNA-binding domain. SHP regulates the expression of several key genes within gluconeogenesis (e.g. phosphoenolpyruvate carboxykinase 1 (PEPCK1)) or lipid metabolism (e.g. sterol regulatory element binding protein 1c (SREBP-1c))^8^. Therefore, modulation of FXR may hold therapeutic potential for type 2 diabetes mellitus, dyslipidemia and liver disorders such as non-alcoholic steatohepatitis (NASH)^6,7,9^. Accordingly, the first-in-class FXR agonist 6α-ethyl-CDCA (**2**, 6-ECDCA, INT-747, obeticholic acid, OCA) has gained approval for primary sclerosing cholangitis and is in late-stage clinical trials for NAFLD and NASH underlining the value of FXR as drug target^10,11^. For the receptor’s extraordinary therapeutic potential, FXR drug discovery is intensive and several compounds including descendants of the widely-used reference compound GW4064 (**3**) are succeeding in clinical trials.

Recently, we reported FXR modulatory potency of the kinase inhibitor imatinib (**4**) with low intrinsic efficacy^12^. Since **4** still possesses high therapeutic relevance in the treatment of chronic myeloid leukemia (CML) such side activity seems important especially because it might be connected to anti-diabetic effects that were observed under treatment with **4**^13–15^. In our consecutive *in silico* and *in vitro* screening campaign^12^., **4** significantly transactivated a full-length FXR reporter gene assay at 30 µM concentration and modulated FXR dependant gene expression. Here we report in-depth evaluation of its mode of FXR activation and characterize it as allosteric modulator of FXR with moderate micromolar potency. Furthermore, we prepared a series of close structural analogues and therein identified an optimized allosteric FXR activator with remarkably enhanced potency.

## Results

**Figure 2:**
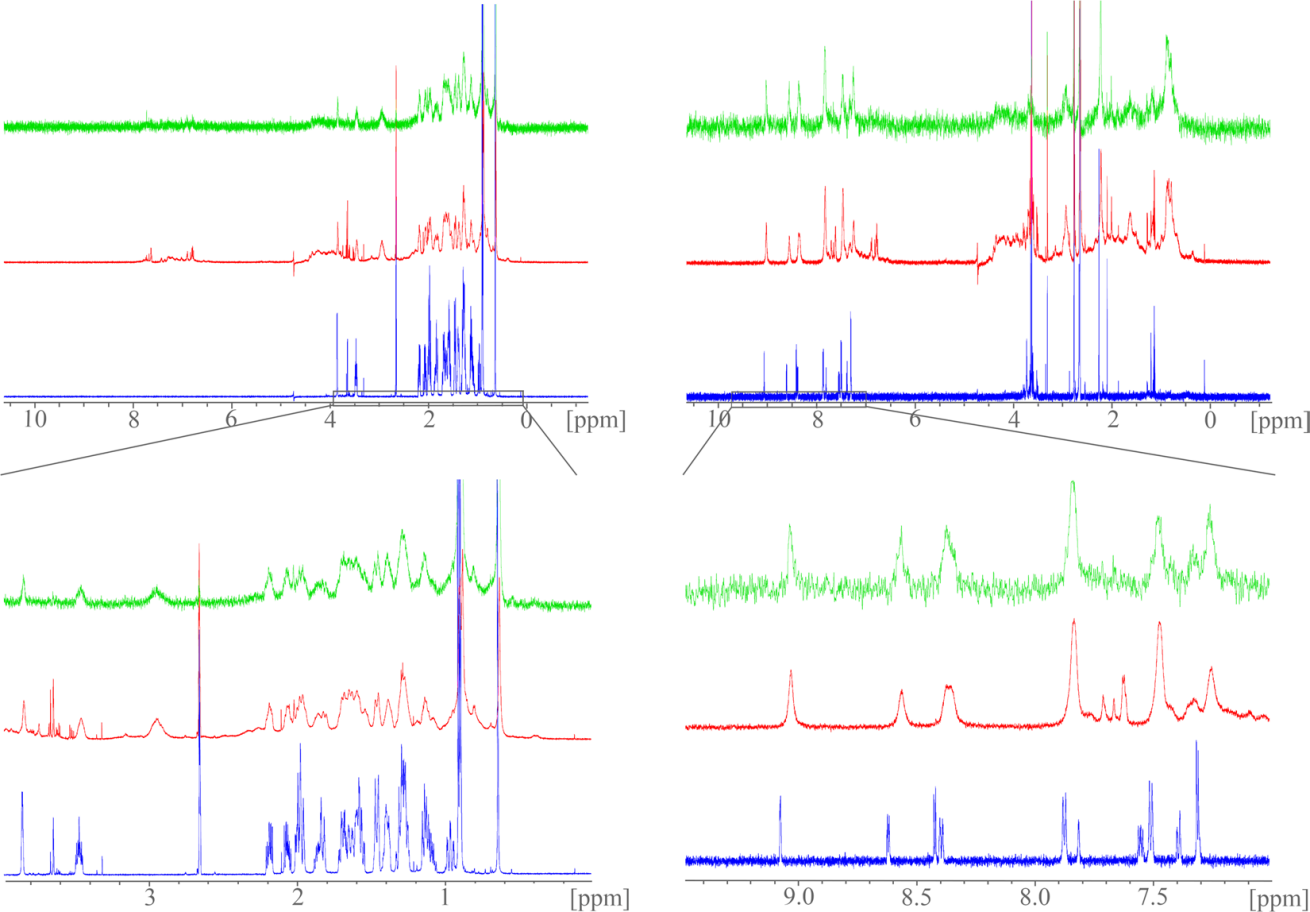
Saturation-transfer difference (STD) NMR experiments confirm binding of **4** to the FXR-LBD: STD-NMR experiments with CDCA (**1**, left spectra) or imatinib (**4**, right spectra) and the FXR-LBD; the upper spectra are covering the full recorded range, the lower magnifications depict the relevant areas; standard ^1^H-NMR spectra of compound alone are depicted in blue, standard ^1^H-NMR spectra in presence of the FXR-LBD are red and STD-NMR spectra are green: ^1^H-NMR signals of **1** and **4** are significantly broadened upon addition of FXR-LBD protein. Additionally, signals of **4** show chemical shift perturbations. When the protein is saturated in the STD experiment (green), magnetization is transferred to **4** indicated by appearance of the same broad aromatic signals of **4** which confirms binding of **4** to the FXR-LBD.

Initial dose-response evaluation of **4** in a full-length FXR transactivation assay revealed an EC_50_-value of 6.6 ± 0.3 µM with a relative maximum activation (rel. max. act.) of 10.7 ± 0.1% compared to FXR full agonist **3** (3 µM) indicating that **4** has partial FXR agonistic activity. To gain evidence that the observed activity is mediated by direct interaction with FXR, we studied binding of imatinib (**4**) to FXR by saturation-transfer difference NMR (STD-NMR) which is particularly suitable to determine binders with low affinity. We recorded NMR spectra of imatinib (**4**) alone and in presence of the FXR ligand binding domain (LBD) as well as an STD spectrum of this mixture (Fig. 2). The endogenous FXR agonist **1** was studied in the same manner for comparison. The standard ^1^H-NMR of **1** and **4** in presence of the FXR-LBD revealed marked line-broadening and chemical shift perturbations especially for the aromatic signals of **4** as indicator for binding. When the FXR-LBD was saturated in the STD-NMR experiment, the same broadened and shifted aromatic signals of **4** became visible and confirmed binding of **4** to the FXR-LBD since magnetization was transferred from protein to ligand.

**Figure 3:**
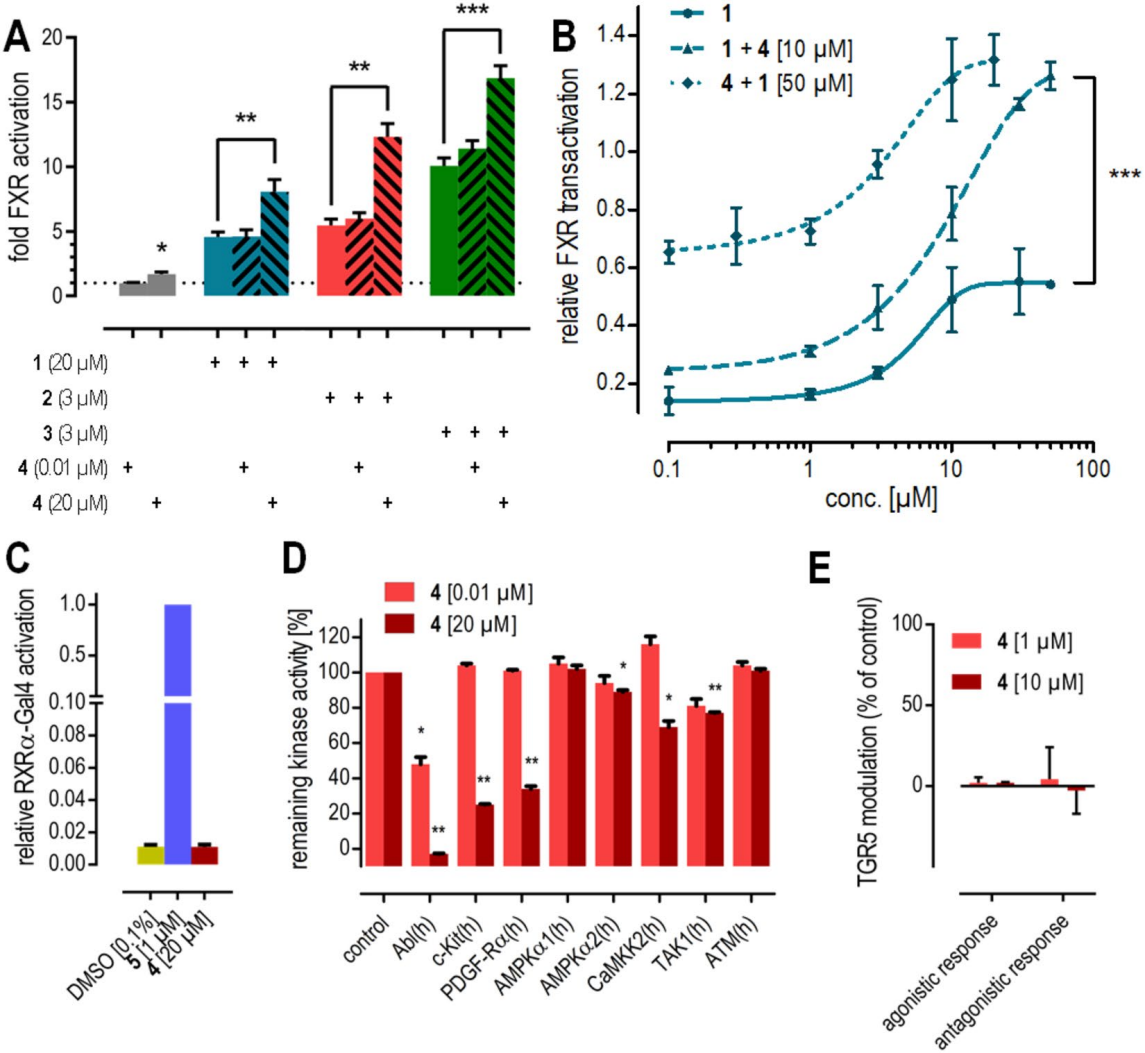
*In vitro* profiling of FXR modulation by **4**. (**A**) At 20 µM, **4** significantly enhances transactivation efficacy of FXR agonists **1**–**3** in a full-length FXR reporter gene assay. (**B**) Dose-response curves of FXR agonist **1** and compound **4** in presence of a fixed concentration of the respective other agent confirms increased maximum relative transactivation without marked changes in EC_50_-values. The maximum relative transactivation exceeds the sum of the single compounds’ maximum relative transactivation suggesting synergy. (**C**) **4** does not activate FXR’s heterodimer partner RXR. (**D**) **4** is a potent inhibitor of the kinases Abl, c-Kit and PDGF-Rα but does not markedly inhibit kinases that interact with FXR. (**E**) **4** does not modulate the membrane bile acid receptor TGR5. *p < 0.05, **p < 0.01, ***p < 0.001.

To evaluate the mode of interaction between **4** and FXR, we studied potential competitive behavior with orthosteric FXR agonists **1**–**3** (Fig. 3A). Expectedly, low concentrations of **4** (0.01 µM) did not affect FXR activation but in a concentration (20 µM) above its EC_50_ value of 6.6 µM, **4** remarkably enhanced FXR activation by the orthosteric ligands instead of reducing FXR-mediated transactivation which would be expected for an orthosteric partial agonist. We studied this effect further by recording dose-response curves of **4** and **1** in presence of a constant concentration of the respective other compound (Fig. 3B). Compared to the dose-response of **1** alone, a constant concentration of 10 µM **4** caused significantly higher maximum relative activation for **1** without markedly affecting its EC_50_ value. Notably, the maximum relative activation markedly exceeded the sum of the maximum relative activation rates of **1** and **4** alone indicating synergy. Oppositely, a constant concentration of 50 µM **1** had the same effect on the dose-response of **4**. Together, these results strongly point to allosteric modulation of FXR by **4**.

To examine whether modulation of proteins interacting with FXR might contribute to the observed effects, we determined the activity of **4** on several other targets known to interact with FXR. Under physiological conditions and in our full-length reporter gene assay, FXR forms permissive heterodimers with the retinoid X receptor that can be activated by agonists of either partner receptor. Thus, **4** was assayed for RXR agonistic activity in a Gal4-DBD-RXRα-LBD hybrid reporter gene assay specifically determining RXR activation (Fig. 3C). However, **4** neither activated the RXR chimera receptor nor competed with the synthetic RXR agonist bexarotene (**5**) excluding that RXR activation contributed to FXR modulatory activity of **4**.

FXR activity can also be modulated by phosphorylation and since **4** is a potent kinase inhibitor, kinase-mediated effects may affect transactivation of the FXR/RXR heterodimer in our reporter gene assay. Therefore, we determined the inhibitory activity of imatinib (**4**) on kinases modifying FXR or RXR in conventional radiometric assays or homogeneous time-resolved fluorescence (HTRF) technology based test systems (Fig. 3D)^16^.

According to literature, a number of kinases can directly or indirectly affect the phosphorylation state of the FXR/RXR-heterodimer^5,17–21^. The most important kinase known to functionally phosphorylate FXR at Ser-250 within the hinge region is AMP-activated protein kinase (AMPK) and AMPK has been characterized as a negative regulator of many FXR target genes^17^. Inhibition of AMPK could therefore potentially cause suppression of this negative regulatory effect leading to FXR activation. However, **4** did not markedly inhibit AMPKα1 or AMPKα2 at 20 µM. Moreover, FXR activity might be affected by the AMPK upstream kinases calcium/calmodulin-dependent protein kinase kinase 2 (CaMKK2), transforming growth factor β activated kinase-1 (TAK1) and ataxia-telangiectasia mutated (ATM) kinase that regulate the activity of AMPK by an activating phosphorylation at Thr-172. Inhibition of these AMPK kinases (AMPKK) might reduce activity of AMPK and consequently reduce inhibitory FXR phosphorylation increasing FXR activity, as well^18,19^. High concentrations of **4** (20 µM) caused slight inhibition of the AMPKKs, CaMKK2 and TAK1, whereas no activity was observed on ATM. Another prominent AMPKK liver kinase B1 (LKB1) is not expressed in HeLa cells in which we studied the FXR activating activity of **4**^19^. Hence, an influence of LKB1 could be excluded. PKC-mediated phosphorylations of FXR could be excluded as well, because **4** containing the flag-methyl-group at position 2 of the central 1,5-diaminophenyl partial structure is inactive at kinases of the PKC-class^5,20^. Additionally, known PKA-mediated phosphorylations of RXR could be excluded, since **4** is known as inactive on PKA^20,21^.

Taken together, it is unlikely that kinase-mediated effects are significantly contributing to FXR modulation by **4**. The observed inhibitory activity of **4** on the AMPKKs, CaMKK2 and TAK1, was very weak especially compared to the efficacy of **4** on its main targets Abl, c-Kit and PDGF-Rα. Cascadic kinase signalling is self-enhancing and known to require strong inhibition for a significant effect. Therefore, enhanced FXR activation by **4** or by **1–3** in presence of **4** does not seem to be affected by kinase inhibition but arise alone from direct modulation of the nuclear receptor.

In addition to FXR, the G-protein coupled receptor takeda G-protein receptor 5 (TGR5, GPCR19) endogenously recognizes bile acids. It was therefore possible that **4** also interacts with this membrane bile acid receptor. *In vitro* evaluation of **4** on TGR5 (Fig. 3E) revealed no agonistic or antagonistic activity, however, confirming selectivity of its allosteric FXR modulatory activity over TGR5.

**Figure 4:**
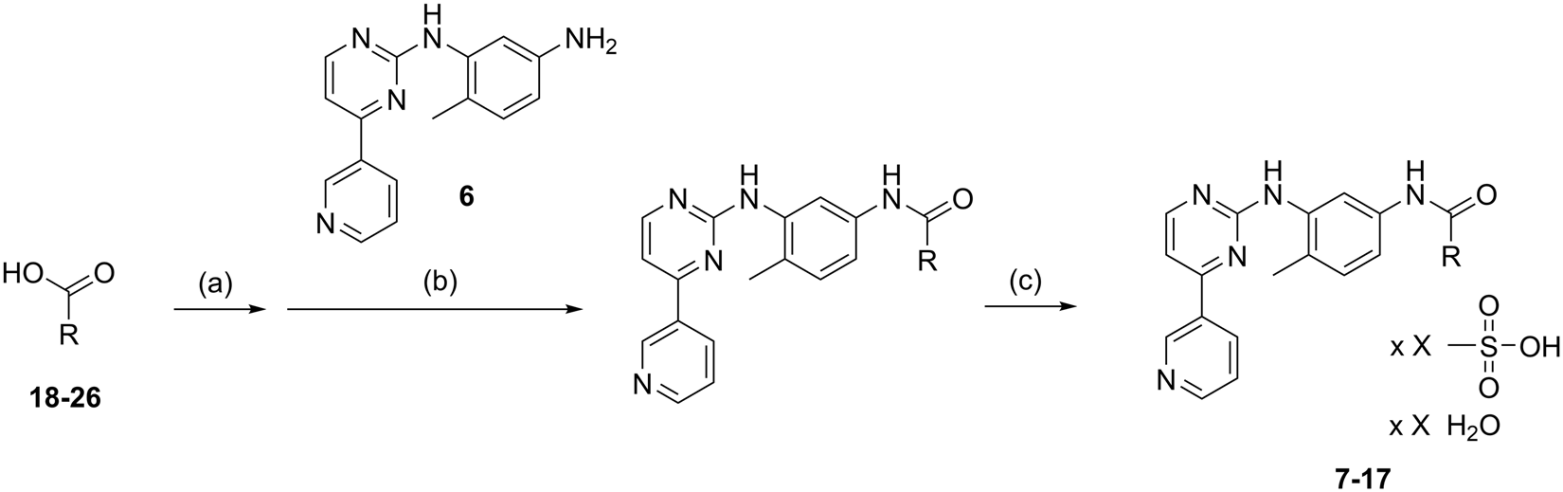
Synthesis of analogues **7**–**17**. Reagents & conditions. (**A**) CDI, DMF, RT, 1 h. (**B**) DMF, 80 °C, 24 h. (**c**) MsOH, MeOH, RT, 1 h.

Ago-positive allosteric modulation of FXR might constitute a very valuable pharmacological strategy since it could enhance the effect of endogenous FXR activating stimuli by natural ligands thereby conserving physiological balance with increased efficacy. To further evaluate this concept, we prepared imatinib analogues **27**–**36** from precursor **6** and carboxylic acids **18**–**26** using CDI in DMF and subsequently transformed them to mesylate salts **7**–**17** for improved solubility and comparability with **4** mesylate (Fig. 4).

As a starting point for structural optimization (Table 1), we characterized the activity of the commercially available imatinib fragment **17** which turned out almost equally active on FXR with an EC_50_ value of 17.8 ± 1.1 µM indicating that the *N*-acyl substituent of **4** poorly contributed to potency and might offer potential for optimization. Therefore, we studied alternative *N*-acyl residues. While acetyl derivative **7** turned out inactive, introduction of a benzoyl moiety in **8** or a 2-naphtoyl moiety in **9** already produced submicromolar FXR modulators. As both compounds were equipotent, we focused on benzoyl derivative **8** due to lower molecular weight and broader substituent diversity and continued our preliminary SAR study with substituted aromatic 6-ring residues. The slightly more polar isonicotinoyl substituent in **10** was less favored while a methyl group in 4-position (**11**) increased potency by almost 10-fold, indicating an optimal substitution site for optimization^22^. When we enlarged the methyl residue of **11** to an ethyl group (**12**), single-digit nanomolar activity was achieved but further enlargement of the lipophilic *para*-substituent to *tert*-butyl (**13**) or phenyl (**14**) caused a significant loss of potency indicating that only small 4-substituents were well tolerated but further optimization potential could lie in the electronic characteristics of the benzoyl residue. Following the Topliss’ strategy^23^, we therefore introduced a 4-methoxy group (**15**) and a 4-chlorine atom (**16**) which showed pronounced effects on potency. Electron releasing methoxy substitution (**15**) was poorly tolerated whereas the electron withdrawing chlorine atom (**16**) further increased activity to an EC_50_-value of 1.9 nM. With this high FXR modulatory potency, **16** seemed sufficiently optimized as potential tool compound mimicking the activity of **4**.

**Table 1:** Structure-activity relationship of 4 and derivatives **7**–**17** on FXR in a full-length reporter gene assay.

#		hFXR: EC_50_ (max. rel. act.)
**4**		6.6 ± 0.3 µM (10.7 ± 0.1%)
**17**		17.8 ± 1.1 µM (9.2 ± 0.1%)
**7**		inactive
**8**		0.45 ± 0.02 µM (9.0 ± 0.1%)
**9**		0.46 ± 0.01 µM (7.1 ± 0.1%)
**10**		3.5 ± 0.2 µM (11.7 ± 0.2%)
**11**		0.051 ± 0.001 µM (8.1 ± 0.1%)
**12**		0.0064 ± 0.0004 µM (7.9 ± 0.1%)
**13**		0.58 ± 0.07 µM (8.6 ± 0.1%)
**14**		0.13 ± 0.02 µM (7.1 ± 0.1%)
**15**		1.0 ± 0.1 µM (8.4 ± 0.1%)
**16**		0.0019 ± 0.0006 µM (8.4 ± 0.2%)

(Data represents mean ± SEM; n ≥ 3; *p* < 0.05 for every concentration ≥ EC_50_ value.) *Salt compositions of **7**–**17** differed and are accurately described in the SI.

**Figure 5:**
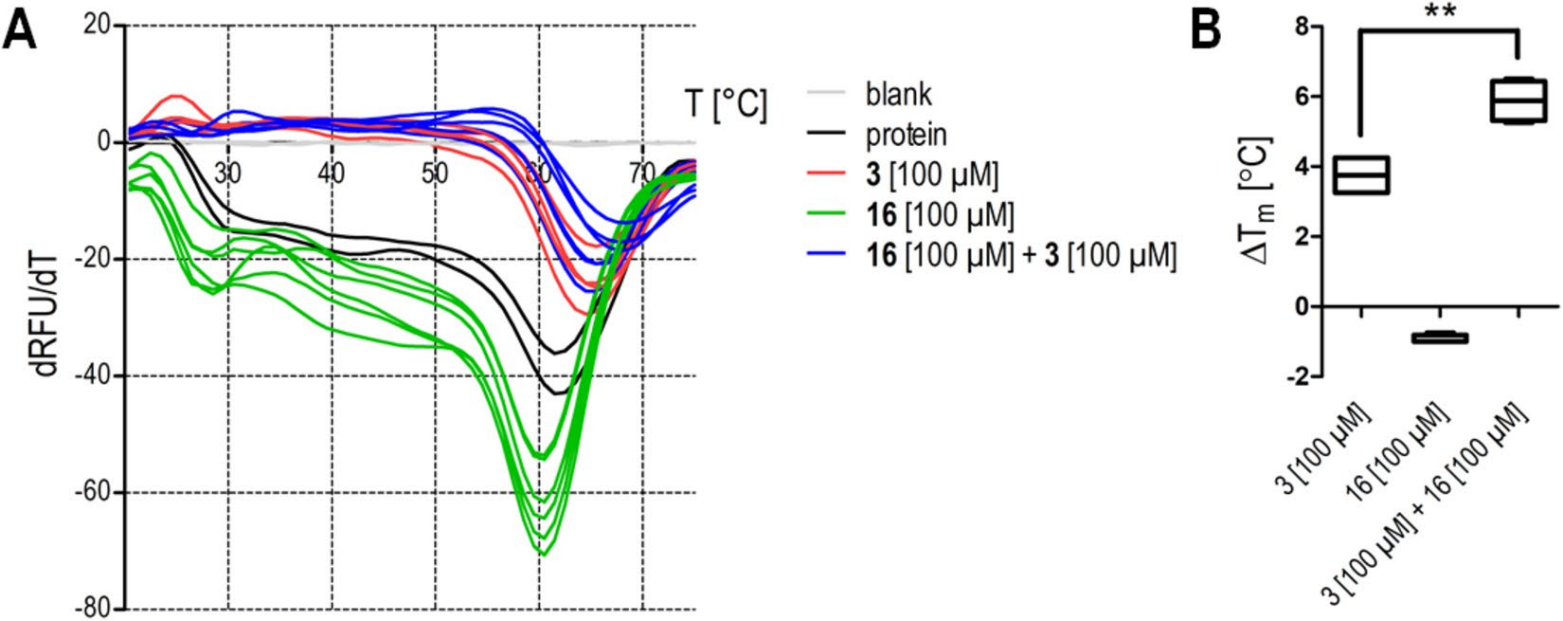
Interaction of **16** with FXR studied by thermal shift. (**A**) Melt curves of the FXR-LBD alone (black), in presence of FXR agonist **3** (red), in presence of **16** (green) and in presence of **3** and **16** together (blue). (SYPRO-Orange, n = 2–6). (**B**) FXR agonist **3** causes a significant thermal shift of the FXR-LBD melting point confirming binding. **16** alone induces a weak negative shift but significantly enhances the thermal shift caused by **3** confirming further FXR-LBD stabilization and a direct interaction.

To confirm similar ago-positive allosteric FXR modulation for optimized analogue **16** as we observed for **4**, **16** was equally characterized *in vitro*. However, STD-NMR is not suitable to detect high-affinity binders because dissociation of magnetized ligand from the protein is required to observe saturation transfer^24,25^. Binding of **16** to the FXR-LBD was therefore confirmed by thermal shift (Fig. 5). **16** alone slightly destabilized the protein causing a negative thermal shift while the combination of **16** and **3** strongly stabilized the LBD with a significantly greater thermal shift than **3** alone. This behaviour also pointed to allosteric binding of **16** which can only promote full activation in presence of an orthosteric agonist.

**Figure 6:**
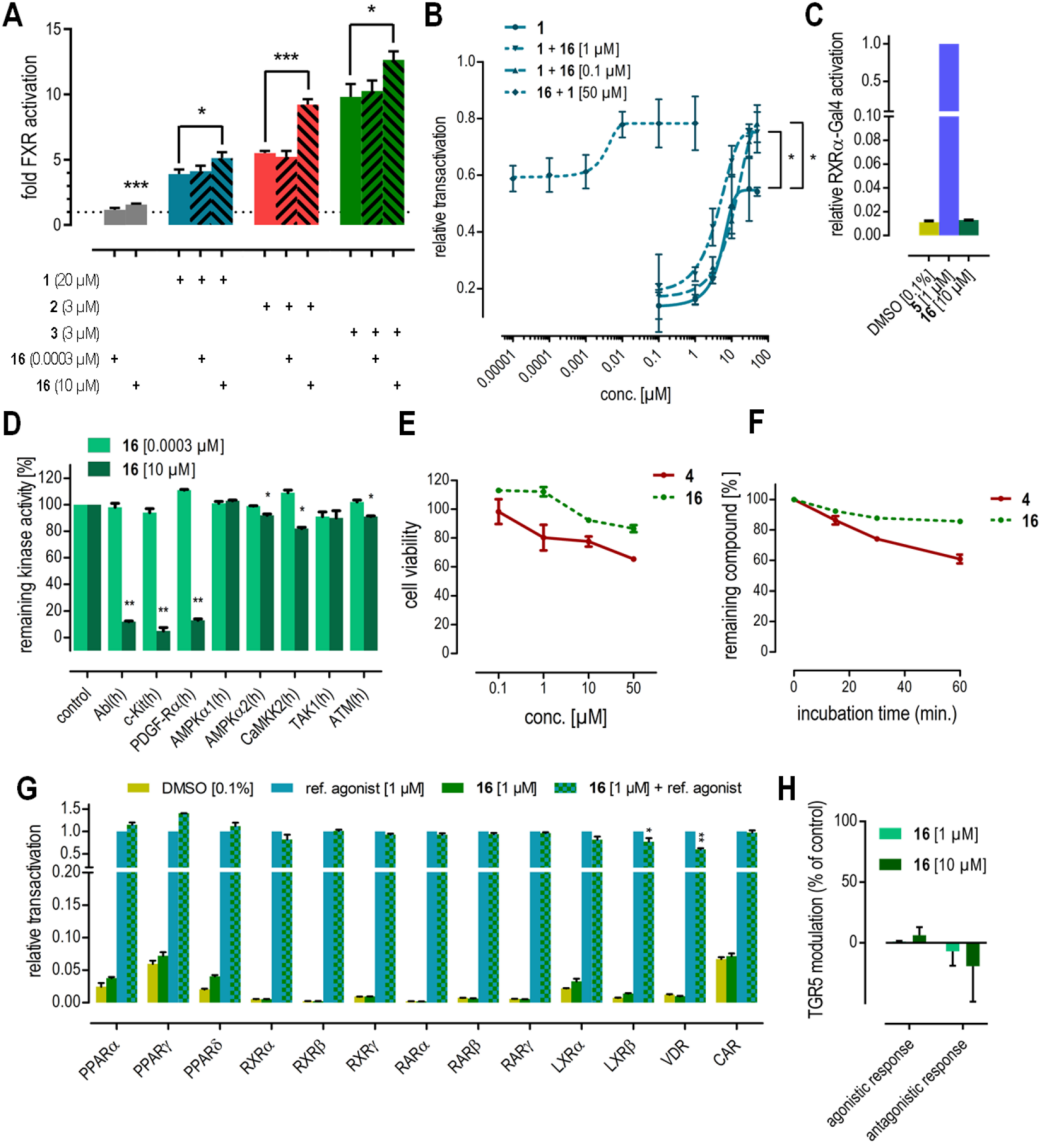
*In vitro* profiling of FXR modulator **16**. (**A**) At 10 µM, **16** significantly enhances transactivation efficacy of FXR agonists **1**–**3** in a full-length FXR reporter gene assay. (**B**) Dose-response curves of FXR agonist **1** and compound **16** in presence of a fixed concentration of the respective other agent confirms increased maximum relative transactivation without marked changes in EC_50_-values. The maximum relative transactivation exceeds the sum of the single compounds’ maximum relative transactivation suggesting synergy. (**C**) **16** does not activate FXR’s heterodimer partner RXR. (**D**) **16** is a potent inhibitor of the kinases Abl, c-Kit and PDGF-Rα but does not markedly inhibit kinases that interact with FXR. (**E**) **16** is less cytotoxic than **4**. (**F**) **16** possesses higher microsomal stability than **4**. (**G**) **16** is highly selective over nuclear receptors related to FXR. (**H**) **16** does not modulate the membrane bile acid receptor TGR5. *p < 0.05, **p < 0.01, ***p < 0.001.

Accordingly, **16** enhanced the FXR activation efficacy of endogenous agonist **1** and the synthetic agonists **2** and **3** in the reporter gene assay (Fig. 6A). The EC_50_-value of **1** was not affected by the presence of 0.1 µM **16** but maximum relative activation increased by about 50% (Fig. 6B). As **4**, **16** did not activate the RXRα-Gal4 chimera receptor nor antagonize its activation by **5** (Fig. 6C) and the kinase inhibition profile of **16** resembled **4** with even less inhibitory activity on the AMPKKs (Fig. 6D). Thus, FXR modulation by **16** is not mediated through FXR’s heterodimer partner RXR nor through kinase inhibition and **16** represents a highly optimized descendant of **4** with allosteric FXR modulatory activity. *In vitro* characterization of **16** revealed significantly lower toxicity (Fig. 6E), enhanced metabolic stability (Fig. 6F) compared to **4** and high selectivity over nuclear receptors related to FXR (Fig. 6G) as well as the membrane bile acid receptor TGR5 (Fig. 6H).

**Figure 7:**
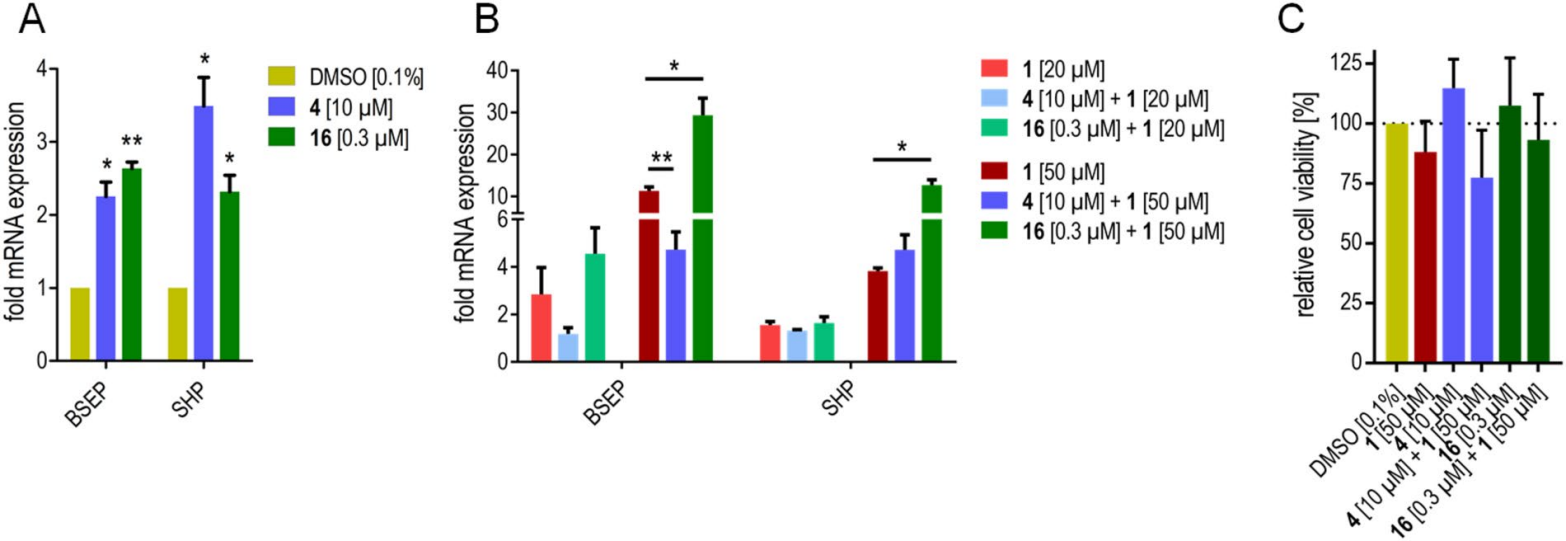
Effects of **4** (10 µM) and **16** (0.3 µM) on FXR mediated gene expression in HepG2 cells in absence (**A**) and presence (**B**) of the endogenous FXR agonist **1** (20 or 50 µM). Observed effects on gene expression are not a result of cytotoxicty (**C**). Data represent mean ± SEM; n ≥ 3; *p < 0.05, **p < 0.01.

To study the cellular effects of allosteric FXR activation, we treated the hepatocarcinoma cell line HepG2 with **4** (10 µM) or **16** (0.3 µM) alone or in presence of **1** (20 or 50 µM) and determined the expression of FXR target genes bile salt export protein (BSEP) and small heterodimer partner (SHP) on mRNA level by quantitative real-time polymerase chain reaction (qRT-PCR, Fig. 7). **4** and **16** alone induced BSEP and SHP expression with low efficacy compared to endogenous agonist **1** (Fig. 7A). When **4** or **16** were co-incubated with endogenous FXR agonist **1**, marked modulatory effects could be observed especially at the higher (50 µM) concentration of **1** (Fig. 7B). **4** strongly diminished the CDCA-induced BSEP expression but caused a trend to additive mRNA induction with respect to SHP. **16** remarkably enhanced CDCA-induced BSEP and SHP expression leading to about 3-fold higher mRNA expression of both genes compared to **1** alone. Toxicity determination of the individual compounds **1**, **4** and **16** and their combinations in the cell line used for gene expression analysis at the highest used concentration revealed no marked toxic activity and confirmed that the observed effects on FXR target gene expression were not an artifact resulting from cytotoxicity (Fig. 7C).

## Discussion

We have recently reported FXR partial agonistic potency of the kinase inhibitor **4** in a full-length FXR reporter gene assay^12^. In this study, we intended to evaluate the mode of FXR modulation by **4** and to develop close analogues with increased potency on FXR. Marked line-broadening, chemical shift perturbation and saturation transfer in our STD-NMR experiments evidence that **4** affects FXR activity via a direct interaction. Since **4** significantly increased the transactivation efficacy of orthosteric FXR agonists **1**–**3**, partial agonistic FXR modulation by **4** is not a result of orthosteric binding. In presence of an orthosteric full agonist, an orthosteric partial agonist would displace the agonists from the binding site and behave as partial antagonist^26^. Involvement of FXR’s heterodimer partner RXR in FXR modulation by **4** can be excluded since no agonistic or antagonistic activity of **4** on a specific Gal4-DBD-RXR-LBD hybrid receptor was observed.

However, nuclear receptor signalling may also be affected by the receptor’s phosphorylation state and especially AMPK is known to modify FXR activity by phosphorylation. Involvement of AMPK signalling in the FXR modulation by **4** cannot be entirely excluded since **4** revealed weak though statistically significant inhibitory potency on AMPKα2 and the AMPKKs CaMKK2 and TAK1. In light of the high remaining activity of all affected kinases the contribution of reduced kinase signalling to FXR modulation by **4** seems very small at best since kinase signalling is self-enhancing and requires robust inhibition for effects.

Intrigued by the potentially novel mode of FXR modulation by **4**, we generated a small series of close analogues to evaluate whether allosteric FXR activation by the compound class can be optimized. Compound **16** comprising a chlorine atom instead of the piperazinyl moiety contained in **4** turned out as strongly optimized allosteric FXR modulator. **16** directly interacts with FXR as evidenced by thermal shift and behaved equally *in vitro* as **4** synergistically enhancing the transactivation efficacy of FXR agonists **1**–**3**. Compound **16** turned out less toxic and more metabolically stable than **4** and was highly selective amongst nuclear receptors but still constitutes a potent inhibitor of the kinases Abl, c-Kit and PDGF-Rα. Thus, it is not suitable for further development but might serve as tool compound.

Allosteric FXR modulators **4** and **16** alone enhanced expression of FXR target genes BSEP and SHP but with low efficacy. In contrast, both compounds strongly affected CDCA (**1**)-induced SHP and BSEP expression. Surprisingly, **4** markedly diminished whereas **16** strongly promoted CDCA (**1**)-induced BSEP expression. Regarding SHP expression, **4** and **16** both enhanced CDCA (**1**)-induced expression although with different efficacy. Such gene-selective activity might be due to differential recruitment of distinct co-activators as it has been described e.g. for some selective PPAR modulators^27^. Our results indicate that FXR activity can be modulated via an allosteric mechanism in both, synergistic ago-positive and allosteric antagonistic fashion. Moreover, minor structural changes seem capable of affecting the mode of allosteric modulation since exchanging the *N*-methylpiperazinylmethyl moiety of **4** by a chlorine atom in **16** changed the allosteric antagonism that **4** exhibits on BSEP expression to synergistic ago-positive activity. Understanding of the structural basis for this gene selective allosteric modulation requires further studies but will be of great value for the development of safer and more efficacious FXR targeting agents.

The effect of **4** on SHP mRNA levels in presence of CDCA (**1**) provides another potential link between the low partial agonistic potency of **4** and significant effects on glucose homeostasis that were observed under treatment with **4**. Physiologically, bile acids such as **1** govern FXR activity and our results indicate that **4** and **16** are capable of enhancing their FXR agonistic activity. Consequently, the expression of SHP is strongly increased and by SHP-dependent repression of PEPCK1 and G-6-Pase, gluconeogenesis is reduced. Altogether, this mechanism provides a possible new explanation for reduced glucose levels upon imatinib treatment and in light of an EC_50_-value of 6.6 ± 0.3 µM for FXR activation, reported plasma levels for **4** of up to 8.1 µM are sufficient to cause FXR modulation under treatment with **4**^6,14,15,28^.

In conclusion, we have classified **4** and descendant **16** as allosteric modulators of FXR^29,30^. This novel mode of FXR modulation might hold considerable pharmacological potential by fueling the efficacy of endogenous FXR agonists. Additionally, distinctly pronounced additive effects on the expression of different genes suggest that allosteric FXR modulation is feasible and could open a new avenue towards gene-selective FXR modulation. Still, allosteric FXR modulators without kinase inhibitory potency are required to promote this concept towards clinical development.

## Experimental Procedures

4-Chloro-*N*-(4-methyl-3-(4-(pyridin-3-yl)pyrimidin-2-ylamino)phenyl)benzamide (**36**): Preparation according to general procedure (**a**) using 4-chlorobenzoic acid (**26**). Yield: 19%. ^1^H-NMR (500 MHz, (CD_3_)_2_SO): *δ* = 10.28 (s, 1H, N*H*-CO), 9.28 (d, *J* = 2.2 Hz, 1H, Pyrid-2-*H*), 8.99 (s, 1H, Pyrim-N*H*), 8.69 (dd, *J* = 4.8 Hz, 1.6 Hz, 1H, Pyrid-4-*H*), 8.52 (d, *J* = 5.1 Hz, 1H, Pyrim-6-*H*), 8.49 (dt, *J* = 8.1 Hz, 1.8 Hz, 1H, Pyrid-6-*H*), 8.08 (d, *J* = 2.0, 1H, Phen-NH-6-*H*), 7.99 (d, *J* = 8.6 Hz, 2H, Phen-CO-2,6-*H*), 7.61 (d, *J* = 8.7 Hz, 2H, Phen-CO-3,5-*H*), 7.53 (dd, *J* = 8.0 Hz, 4.2 Hz, 1H, Pyrid-5-*H*), 7.48 (dd, *J* = 8.2 Hz, 2.1 Hz, 1H, Phen-NH-4*H*), 7.44 (d, *J* = 5.2 Hz, 1H, Pyrim-5-*H*), 7.22 (d, *J* = 8.4, Phen-NH-3*H*), 2.23 (s, 3H, -C*H*_3_); ^13^C-NMR (125.77 MHz, (CD_3_)_2_SO) *δ*: 164.71 (*C*=O), 162.08 (Pyrim-4-*C*), 161.62 (Pyrim-2-*C*), 159.96 (Pyrim-6-*C*), 151.88 (Pyrid-4-*C*), 148.67 (Pyrid-2-*C*), 138.30 (Phen-NH-1-*C*), 137.41 (Phen-NH-5-*C*), 136.75 (Phen-CO-4-*C*), 134.90 (Pyrid-6-*C*), 134.22 (Phen-CO-1-*C*), 132.66 (Pyrid-1-*C*), 130.55 (Phen-NH-3-*C*), 130.06 (Phen-CO-2,6-*C*), 128.92 (Phen-CO-3,5-*C*), 128.27 (Phen-NH-2-*C*), 124.27 (Pyrid-5-*C*), 117.68 (Phen-NH-6-*C*), 117.21 (Phen-NH-4-*C*), 108.03 (Pyrim-5-*C*), 18.14 (-*C*H_3_); R_f_ (PE/THF = 1/1): 0.17; C_23_H_18_ClN_5_O; MS (ESI+): m/z = 416.1 [M + H]^+^; colourless solid^1,2^.

4-Chloro-*N*-(4-methyl-3-(4-(pyridin-3-yl)pyrimidin-2-ylamino)phenyl)benzamide dimesylate-sesquihydrate (**16**): Preparation according to general procedure (**b**) using **36**. Yield: 43%. ^1^H-NMR (500 MHz, (CD_3_)_2_SO): *δ* = 10.29 (s, 1H, N*H*-CO), 9.38 (d, *J* = 1.8 Hz, 1H, Pyrid-2-*H*), 9.09 (s, 1H, Pyrim-N*H*), 8.82 (dd, *J* = 5.3 Hz, 1.3 Hz, 1H, Pyrid-4-*H*), 8.77 (dt, *J* = 8.1 Hz, 1.8 Hz, 1H, Pyrid-6-*H*), 8.58 (d, *J* = 6.2 Hz, 1H, Pyrim-6-*H*), 8.12 (d, *J* = 1.7, 1H, Phen-NH-6-*H*), 8.00 (d, *J* = 8.6 Hz, 2H, Phen-CO-2,6-*H*), 7.78 (dd, *J* = 8.0 Hz, 4.2 Hz, 1H, Pyrid-5-*H*), 7.62 (d, *J* = 8.7 Hz, 2H, Phen-CO-3,5-*H*), 7.51 (d, *J* = 5.2 Hz, 1H, Pyrim-5-*H*), 7.46 (dd, *J* = 8.2 Hz, 2.1 Hz, 1H, Phen-NH-4*H*), 7.23 (d, *J* = 8.4, Phen-NH-3*H*), 2.32 (s, 6H, S-C*H*_3_), 2.24 (s, 3H, -C*H*_3_); ^13^C-NMR (125.77 MHz, (CD_3_)_2_SO) *δ*: 164.75 (*C*=O), 161.47 (Pyrim-2-*C*), 160.28 (Pyrim-6-*C*), 160.17 (Pyrim-4-*C*), 149.15 (Pyrid-4-*C*), 146.22 (Pyrid-2-*C*), 138.20 (Pyrid-6-*C*), 137.70 (Phen-NH-1-*C*), 137.39 (Phen-NH-5-*C*), 136.78 (Phen-CO-4-*C*), 134.18 (Phen-CO-1-*C*), 133.86 (Pyrid-1-*C*), 130.73 (Phen-NH-3-*C*), 130.20 (Phen-CO-2,6-*C*), 129.02 (Phen-CO-3,5-*C*), 128.25 (Phen-NH-2-*C*), 125.59 (Pyrid-5-*C*), 117.78 (Phen-NH-6-*C*), 117.42 (Phen-NH-4-*C*), 108.28 (Pyrim-5-*C*), 40.24 (S-*C*H_3_), 18.10 (-*C*H_3_); C_23_H_18_ClN_5_O × 2 CH_4_O_3_S × 1.5 H_2_O; combustion analysis: measured (calculated): C 47.36 (47.28); H 4.40 (4.60); N 11.28 (11.03); orange solid.

For general synthetic procedures and analytical characterization of **7**–**15** as well as their precursors, please refer to supporting information.

### Full-length FXR transactivation assay

#### Plasmids

pcDNA3-hFXR contains the sequence of human FXR and was already published elsewhere^12^. pGL3basic (Promega Corporation, Fitchburg, WI, USA) was used as a reporter plasmid, with a shortened construct of the promotor of the bile salt export protein (BSEP) cloned into the SacI/NheI cleavage site in front of the luciferase gene^31^. pRL-SV40 (Promega) was transfected as a control for normalization of transfection efficiency and cell growth. pSG5-hRXR was already published elsewhere as well^32^.

#### Assay procedure

HeLa cells were grown in DMEM high glucose supplemented with 10% FCS, sodium pyruvate (1 mM), penicillin (100 U/mL) and streptomycin (100 µg/mL) at 37 °C and 5% CO_2_. 24 h before transfection, HeLa cells were seeded in 96-well plates with a density of 8000 cells per well. 3.5 h before transfection, medium was changed to DMEM high glucose, supplemented with sodium pyruvate (1 mM), penicillin (100 U/mL), streptomycin (100 µg/mL) and 0.5% charcoal-stripped FCS. Transient transfection of HeLa cells with BSEP-pGL3, pRL-SV40 and the expression plasmids pcDNA3-hFXR and pSG5-hRXR was carried out using calcium phosphate transfection method. 16 h after transfection, medium was changed to DMEM high glucose, supplemented with sodium pyruvate (1 mM), penicillin (100 U/mL), streptomycin (100 µg/mL) and 0.5% charcoal-stripped FCS. 24 h after transfection, medium was changed to DMEM without phenol red, supplemented with sodium pyruvate (1 mM), penicillin (100 U/mL), streptomycin (100 µg/mL), L-glutamine (2 mM) and 0.5% charcoal-stripped FCS, now additionally containing 0.1% DMSO and the respective test compound or 0.1% DMSO alone as untreated control. Each concentration was tested in triplicate wells and each experiment was repeated independently at least three times. Following 24 h incubation with the test compounds, cells were assayed for luciferase activity using Dual-Glo™ Luciferase Assay System (Promega) according to the manufacturer’s protocol. Luminescence was measured with a Tecan Infinite M200 luminometer (Tecan Deutschland GmbH, Crailsheim, Germany). Normalization of transfection efficiency and cell growth was done by division of firefly luciferase data by renilla luciferase data multiplied by 1000 resulting in relative light units (RLU). Fold activation was obtained by dividing the mean RLU of the tested compound at a respective concentration by the mean RLU of untreated control. Relative activation was obtained by dividing the fold activation of the tested compound at a respective concentration by the fold activation of FXR full agonist **3** at 3 µM. EC_50_ and standard error of the mean values were calculated with the mean relative activation values of at least three independent experiments by SigmaPlot 10.0 (Systat Software GmbH, Erkrath, Germany) using a four-parameter logistic regression. The assay was validated with FXR agonists **1** (EC_50_ = 18 ± 1 µM, 88 ± 3% rel. max. act.), **2** (EC_50_ = 0.16 ± 0.02 µM, 87 ± 3% rel. max. act.) and **3** (EC_50_ = 0.51 ± 0.16 µM, 3 µM defined as 100%)^33^.

### Hybrid reporter gene assays for PPARα/γ/δ, LXRα/β, RXRα/β/γ, RARα/β/γ, VDR and CAR

#### Plasmids

The Gal4-fusion receptor plasmids pFA-CMV-hPPARα-LBD^34^, pFA-CMV-hPPARγ-LBD^34^, pFA-CMV-hPPARδ-LBD^34^, pFA-CMV-hLXRα-LBD^35^, pFA-CMV-hLXRβ-LBD^35^, pFA-CMV-hRXRα-LBD^36^, pFA-CMV-hRXRβ-LBD^36^, pFA-CMV-hRXRγ-LBD^36^, pFA-CMV-hRARα-LBD^36^, pFA-CMV-hRARβ-LBD^36^, pFA-CMV-hRARγ-LBD^36^, pFA-CMV-hVDR-LBD^36^ and pFA-CMV-hCAR-LBD^36^ coding for the hinge region and ligand binding domain (LBD) of the canonical isoform of the respective nuclear receptor have been reported previously. pFR-Luc (Stratagene) was used as reporter plasmid and pRL-SV40 (Promega) for normalization of transfection efficiency and cell growth.

#### Assay procedure

HEK293T cells were grown in DMEM high glucose, supplemented with 10% FCS, sodium pyruvate (1 mM), penicillin (100 U/mL) and streptomycin (100 μg/mL) at 37 °C and 5% CO_2_. The day before transfection, HEK293T cells were seeded in 96-well plates (2.5·10^4^ cells/well). Before transfection, medium was changed to Opti-MEM without supplements. Transient transfection was carried out using Lipofectamine LTX reagent (Invitrogen) according to the manufacturer’s protocol with pFR-Luc (Stratagene), pRL-SV40 (Promega) and pFA-CMV-hNR-LBD. 5 h after transfection, medium was changed to Opti-MEM supplemented with penicillin (100 U/mL), streptomycin (100 μg/mL), now additionally containing 0.1% DMSO and the respective test compound or 0.1% DMSO alone as untreated control. Each concentration was tested in duplicates and each experiment was repeated independently at least three times. Following overnight (12–14 h) incubation with the test compounds, cells were assayed for luciferase activity using Dual-Glo™ Luciferase Assay System (Promega) according to the manufacturer’s protocol. Luminescence was measured with an Infinite M200 luminometer (Tecan Deutschland GmbH) or a Tecan Spark 10 M luminometer (Tecan). Normalization of transfection efficiency and cell growth was done by division of firefly luciferase data by renilla luciferase data and multiplying the value by 1000 resulting in relative light units (RLU). Fold activation was obtained by dividing the mean RLU of a test compound at a respective concentration by the mean RLU of untreated control. Relative activation was obtained by dividing the fold activation of a test compound at a respective concentration by the fold activation of a respective reference agonist at 1 µM (PPARα: GW7647; PPARγ: pioglitazone; PPARδ: L165,041; LXRα/β: T0901317; RXRs: bexarotene; RARs: tretinoin; VDR: calcitriol; CAR: CITCO). All hybrid assays were validated with the above-mentioned reference agonists which yielded EC_50_ values in agreement with literature.

### STD-NMR experiments

STD NMR experiments were performed in STD-NMR buffer (D_2_O with 10 mM Tris-d11 (DCl), 100 mM NaCl, 2 mM DTT-d10, pD = 8.3). The storage buffer of the purified FXR-LBD protein was exchanged against STD-NMR buffer by 10 cycles of diluting the protein solution with STD-NMR buffer 1:2, before concentrating it to the starting volume via Vivaspin2 5 kDa MWCO centricons (GE Healthcare). STD NMR samples were prepared in a 550 µL scale with 50 µM FXR-LBD (or STD-NMR buffer in case of the reference measurement of the test compounds) and 1.5% DMSO-*d*_*6*_ with either **1** (500 µM final concentration) or **4** (500 µM final concentration). Samples were measured with a 700 MHz Bruker AvIIIHD spectrometer equipped with a ^1^H{^31^P/^13^C/^15^N} cryogenic quadruple-resonance probe (QCI) at 298 K. Pulse programs, described by A. D. Gossert and W. Jahnke were downloaded from the Bruker database and adjusted for the 700 MHz Bruker AvIIIHD spectrometer^37^. To suppress the residual water resonances the excitation sculpting method was used in all measurements. For STD-experiments the frequencies for ON and OFF irradiation were set at +0.5 and −30 ppm saturation for 2 s using a 50-ms Gaussian pulse train (10 µW) truncated at 1% with a 50 ms spin-lock to suppress protein signals (CDCA: scans = 512, relaxation delay = 3 s and acquisition time = 1.5 s. Imatinib: scans = 1024, relaxation delay = 3 s and acquisition time = 0.5 s). The standard 1D ^1^H-NMR experiments were performed with a perfect echo sequence with the following parameters (scans = 128, relaxation delay = 1 s and acquisition time = 1.95 s).

### Thermal shift assay

Thermal shift experiments were performed in clear 96-well plates (Invitrogen) using SYPRO Orange (Invitrogen) as dye as described previously^38^. 10 μL of test compound (final concentrations: **3**: 100 µM; **16**: 100 µM) in assay buffer (10 mM TRIS (pH 8.3), 5 mM DTT, 0.5 mM EDTA, 100 mM NaCl) were mixed with 10 μL of FXR-LBD (final protein concentration 5 µM) in assay buffer and 5 µL of SYPRO Orange (5 × final concentration). Temperature-dependent fluorescence increase reporting protein denaturation was measured in duplicates in an ICycler (Bio-Rad Laboratories, Hercules, CA, USA) from 20 to 90 °C in steps of 0.5 °C per minute at 300 nm excitation and 570 nm emission wavelength. The first derivative of the melting curve was calculated using the Graph Pad Prism 5 software. All curves were determined at least four times in independent experiments. Results (expressed as mean ± SEM; n ≥ 4): **3**: 100 µM: 3.75 ± 0.25 °C; **16**: 100 µM: −1.0 ± 0.0 °C; **3**: 100 µM + **16**: 100 µM: 6.00 ± 0.33 °C.

### FXR target gene quantification (quantitative real-time PCR)

FXR target gene quantification was performed as described previously^33,39^. In brief, HepG2 cells were incubated with test compound **4** (10 µM), **16** (0.3 µM) or **1** (20 or 50 µM), or 0.1% DMSO alone as untreated control for 24 h, harvested, washed with cold phosphate buffered saline (PBS) and then directly used for RNA extraction. Two micrograms of total RNA were extracted from HepG2 cells by the Total RNA Mini Kit (R6834-02, Omega Bio-Tek, Inc., Norcross, GA, USA). RNA was reverse-transcribed into cDNA using the High-Capacity cDNA Reverse Transcription Kit (4368814, Thermo Fischer Scientific, Inc.) according to the manufacturer’s protocol. FXR target gene expression was evaluated by quantitative real time PCR analysis with a StepOnePlus™ System (Life Technologies, Carlsbad, CA, USA) using PowerSYBRGreen (Life Technologies; 12.5 µL per well). The primers have been described previously^33,39^. Each sample was set up in duplicates and repeated in at least three independent experiments. The expression was quantified by the comparative ∆∆Ct method and glycerinealdehyde 3-phosphate dehydrogenase (GAPDH) served as reference gene. Results (expressed as mean fold activation ± SEM compared to DMSO (0.1%); n ≥ 3): BSEP: **1** (20 µM): 2.85 ± 0.80; **1** (50 µM): 11.32 ± 0.93; **4** (10 µM): 2.25 ± 0.16; **16** (0.3 µM): 2.64 ± 0.07; **1** (20 µM) + **4** (10 µM): 1.19 ± 0.18; **1** (20 µM) + **16** (0.3 µM): 4.56 ± 0.77; **1** (50 µM) + **4** (10 µM): 4.73 ± 0.61; **1** (50 µM) + **16** (0.3 µM): 29.37 ± 3.32. SHP: **1** (20 µM): 1.56 ± 0.12; **1** (50 µM): 3.83 ± 0.14; **4** (10 µM): 3.49 ± 0.32; **16** (0.3 µM): 2.32 ± 0.18; **1** (20 µM) + **4** (10 µM): 1.33 ± 0.04; **1** (20 µM) + **16** (0.3 µM): 1.64 ± 0.23; **1** (50 µM) + **4** (10 µM): 4.72 ± 0.52; **1** (50 µM) + **16** (0.3 µM): 12.70 ± 1.04.

## Electronic supplementary material

Supporting Information
