# Supplementary material for: Allosteric modulation of the farnesoid X receptor by a small molecule
Source: Sci Rep. 2018 May 1;8:6846. doi: 10.1038/s41598-018-25158-5 (PMC5931576; doi:10.1038/s41598-018-25158-5)
Supplement: Supplementary file 1 — Supporting Information [file 41598_2018_25158_MOESM1_ESM.pdf]

Allosteric modulation of the farnesoid X receptor by a small molecule

Matthias Gabler[a], Jan Kramer[a], Jurema Schmidt[a], Julius Pollinger[a], Julia Weber[a], Astrid Kaiser[a], Frank Löhr[b], Ewgenij Proschak[a], Manfred Schubert-Zsilavecz[a], and Daniel Merk[a*]

[a] Institute of Pharmaceutical Chemistry, Goethe-University Frankfurt, Max-von-Laue-Str. 9, D-60438 Frankfurt, Germany.

[b] Institute of Biophysical Chemistry, Goethe-University Frankfurt, Max-von-Laue-Straße 9, D-60438 Frankfurt, Germany

* merk@pharmchem.uni-frankfurt.de

- SUPPORTING INFORMATION -

Table of Contents

Chemistry 2

In vitro biological evaluation 12

Chemistry

General

All chemicals were purchased from Sigma-Aldrich, Alfa Aesar or Acros Organics and were used without further purification. Analytical TLC (thin layer chromatography) was performed with TLC plates F254 (Merck, Darmstadt, Germany) with detection using a UV-lamp. All NMR spectra were recorded on a Bruker AV 500 spectrometer (Bruker Corporation, Billerica, MA, USA). Chemical shifts (δ) are reported in ppm relative to tetramethylsilane (TMS) as reference; multiplicity: s, singlet; d, doublet; dd, doublet of doublets; t, triplet; dt, doublet of triplets; m, multiplet; approximate coupling constants (J) are shown in hertz (Hz). Mass spectra were obtained on a VG Platform II (Thermo Fischer Scientific, Inc., Waltham, MA, USA) using electrospray ionization (ESI). Elemental analyses (C, H, N) were measured on a Vario MicroCube (Heraeus Holding GmbH, Hanau, Germany) and were within ± 0.4% of the theoretical values for all final compounds, which corresponds to ≥ 95% purity.

Synthesis

Preparation of imatinib derivatives (free bases) **27-36**

General procedure (**a**) for amide synthesis

The respective carboxylic acid **17-26** (1.0 eq.) and *N,N'*-carbonyldiimidazole (1.0 eq.) were dissolved in dry *N,N*-dimethylformamide (DMF, 3 mL/mmol **17-26**). The colourless solution was stirred for 1 h at room temperature. After complete conversion of the carboxylic acid monitored by TLC, 6-methyl-N^1-(4-(pyridin-3-yl)pyrimidin-2-yl)benzene-1,3-diamine (**6**, 1.0 eq.) was added resulting in a yellow solution. After stirring for 24 h at 80 °C, the crude product was precipitated by addition of water and isolated by filtration. The crude product was washed with water and dried at room temperature and atmospheric pressure. For purification, column chromatography was used (mobile phase gradient: freshly distilled petroleum ether (PE) / tetrahydrofurane (THF) starting with 100% PE, ending with 50% PE). Finally, the purified product was dissolved in THF and was precipitated again by addition of n-pentane. The solid purified product (**27-36**) was isolated by filtration, washed with n-pentane and dried at 80 °C and atmospheric pressure.

N-(4-methyl-3-(4-(pyridin-3-yl)pyrimidin-2-ylamino)phenyl)acetamide (**27**)

Preparation according to general procedure (**a**) using acetic acid. Yield: 27%

^{1}H-NMR (500 MHz, $(CD_3)_2SO$): *δ* = 9.86 (s, 1H, N*H*-CO), 9.26 (d, *J* = 2.2 Hz, 1H, Pyrid-2-*H*), 8.93 (s, 1H, Pyrim-N*H*), 8.70 (dd, *J* = 4.8 Hz, 1.6 Hz, 1H, Pyrid-4-*H*), 8.51 (d, *J* = 5.1 Hz, 1H, Pyrim-6-*H*), 8.45 (dt, *J* = 8.1 Hz, 1.8 Hz, 1H, Pyrid-6-*H*), 7.85 (d, *J* = 2.0, 1H, Phen-NH-6-*H*), 7.54 (dd, *J* = 8.0 Hz, 4.2 Hz, 1H, Pyrid-5-*H*), 7.43 (d, *J* = 5.2 Hz, 1H, Pyrim-5-*H*), 7.29 (dd, *J* = 8.2 Hz, 2.1 Hz, 1H, Phen-NH-4*H*), 7.14 (d, *J* = 8.4, Phen-NH-3*H*), 2.18 (s, 3H, Phen-CH_3), 2.03 (s, 3H, H_3C-CO); ^{13}C-NMR (125.77 MHz, $(CD_3)_2SO$) *δ*: 168.50 (*C*=O), 162.07 (Pyrim-4-*C*),

161.60 (Pyrim-2-*C*), 159.92 (Pyrim-6-*C*), 151.85 (Pyrid-4-*C*), 148.64 (Pyrid-2-*C*), 138.26 (Phen-NH-1-*C*), 137.80 (Phen-NH-5-*C*), 134.86 (Pyrid-6-*C*), 132.67 (Pyrid-1-*C*), 130.56 (Phen-NH-3-*C*), 127.35 (Phen-NH-2-*C*), 124.29 (Pyrid-5-*C*), 116.35 (Phen-NH-6-*C*), 115.86 (Phen-NH-4-*C*), 107.97 (Pyrim-5-*C*), 24.47 (H_3C-CO), 18.06 (Phen-CH_3); R_f (THF): 0.34; $C_{18}H_{17}N_5O$; MS (ESI +): m/z = 320.6 $[M+H]^+$; colourless solid.

N-(4-Methyl-3-(4-(pyridin-3-yl)pyrimidin-2-ylamino)phenyl)benzamide (**28**)

Preparation according to general procedure (**a**) using benzoic acid (**18**). Yield: 35%

^{1}H-NMR (500 MHz, $(CD_3)_2SO$): δ = 10.22 (s, 1H, N*H*-CO), 9.29 (d, *J* = 2.2 Hz, 1H, Pyrid-2-*H*), 8.99 (s, 1H, Pyrim-N*H*), 8.69 (dd, *J* = 4.8 Hz, 1.6 Hz, 1H, Pyrid-4-*H*), 8.52 (d, *J* = 5.1 Hz, 1H, Pyrim-6-*H*), 8.49 (dt, *J* = 8.1 Hz, 1.8 Hz, 1H, Pyrid-6-*H*), 8.09 (d, *J* = 2.0, 1H, Phen-NH-6-*H*), 7.95 (d, *J* = 7.0 Hz, 2H, Phen-CO-2,6-*H*), 7.61-7.57 (m, 1H, Phen-CO-4-*H*), 7.55-7.51 (m, 2H, Phen-CO-3,5-*H*), 7.53 (dd, *J* = 8.0 Hz, 4.2 Hz, 1H, Pyrid-5-*H*), 7.49 (dd, *J* = 8.2 Hz, 2.1 Hz, 1H, Phen-NH-4*H*), 7.44 (d, *J* = 5.2 Hz, 1H, Pyrim-5-*H*), 7.21 (d, *J* = 8.4, Phen-NH-3*H*), 2.23 (s, 3H, -CH_3); ^{13}C-NMR (125.77 MHz, $(CD_3)_2SO$) δ: 165.85 (*C*=O), 162.07 (Pyrim-4-*C*), 161.65 (Pyrim-2-*C*), 159.96 (Pyrim-6-*C*), 151.87 (Pyrid-4-*C*), 148.68 (Pyrid-2-*C*), 138.27 (Phen-NH-1-*C*), 137.62 (Phen-NH-5-*C*), 135.57 (Phen-CO-1-*C*), 134.90 (Pyrid-6-*C*), 132.68 (Pyrid-1-*C*), 131.93 (Phen-CO-4-*C*), 130.51 (Phen-NH-3-*C*), 128.84 (Phen-CO-3,5-*C*), 128.12 (Phen-NH-2-*C*), 128.08 (Phen-CO-2,6-*C*), 124.26 (Pyrid-5-*C*), 117.68 (Phen-NH-6-*C*), 117.20 (Phen-NH-4-*C*), 107.99 (Pyrim-5-*C*), 18.10 (-CH_3); R_f (PE/THF = 1/1): 0.18; $C_{23}H_{19}N_5O$; MS (ESI +): m/z = 382.9 $[M+H]^+$; colourless solid.

N-(4-Methyl-3-(4-(pyridin-3-yl)pyrimidin-2-ylamino)phenyl)-2-naphthamide (**29**)

Preparation according to general procedure (**a**) using 2-naphthoic acid (**19**). Yield: 37%

^{1}H-NMR (500 MHz, $(CD_3)_2SO$): δ = 10.40 (s, 1H, N*H*-CO), 9.30 (d, *J* = 2.2 Hz, 1H, Pyrid-2-*H*), 9.01 (s, 1H, Pyrim-N*H*), 8.70 (dd, *J* = 4.8 Hz, 1.6 Hz, 1H, Pyrid-4-*H*), 8.58 (s, 1H, Napht-1-*H*), 8.53 (d, *J* = 5.1 Hz, 1H, Pyrim-6-*H*), 8.50 (dt, *J* = 8.1 Hz, 1.8 Hz, 1H, Pyrid-6-*H*), 8.14 (d, *J* = 2.0, 1H, Phen-NH-6-*H*), 8.09-8.01 (m, 4H, Napht-3,4,5,8-*H*), 7.67-7.61 (m, 2H, Napht-6,7-*H*), 7.55-7.52 (m, 2H, Pyrid-5-*H*, Phen-NH-4*H*), 7.45 (d, *J* = 5.2 Hz, 1H, Pyrim-5-*H*), 7.24 (d, *J* = 8.4, Phen-NH-3*H*), 2.25 (s, 3H, -CH_3); ^{13}C-NMR (125.77 MHz, $(CD_3)_2SO$) δ: 165.89 (*C*=O), 162.08 (Pyrim-4-*C*), 161.66 (Pyrim-2-*C*), 159.98 (Pyrim-6-*C*), 151.88 (Pyrid-4-*C*), 148.69 (Pyrid-2-*C*), 138.31 (Phen-NH-1-*C*), 137.70 (Phen-NH-5-*C*), 134.91 (Pyrid-6-*C*), 134.70 (Napht-4a-*C*), 132.88 (Napht-2-*C*), 132.68 (Pyrid-1-*C*), 132.56 (Napht-8a-*C*), 130.56 (Phen-NH-3-*C*), 129.41 (Napht-8-*C*), 128.46 (Napht-4-*C*), 128.34 (Napht-1-*C*), 128.25 (Napht-6-*C*), 128.14 (Napht-5-*C*), 128.09 (Phen-NH-2-*C*), 127.31 (Napht-7-*C*), 124.97 (Napht-3-*C*), 124.28 (Pyrid-5-*C*), 117.67 (Phen-NH-6-*C*), 117.18 (Phen-NH-4-*C*), 108.01 (Pyrim-5-*C*), 18.15 (-CH_3); R_f (PE/THF = 1/1): 0.19; $C_{27}H_{21}N_5O$; MS (ESI +): m/z = 433.3 $[M+2H]^+$; pale yellow solid.

N-(4-Methyl-3-(4-(pyridin-3-yl)pyrimidin-2-ylamino)phenyl)isonicotinamide (**30**)

Preparation according to general procedure (**a**) using isonicotinic acid (**20**). Yield: 29%

^{1}H-NMR (500 MHz, $(CD_3)_2SO$): δ = 10.47 (s, 1H, N*H*-CO), 9.28 (d, *J* = 2.2 Hz, 1H, Pyrid-2-*H*), 9.00 (s, 1H, Pyrim-N*H*), 8.79 (dd, *J* = 4.5 Hz, 1.6 Hz, 2H, -CO-Pyrid-3,5-*H*), 8.70 (dd, *J* = 4.8 Hz, 1.6 Hz, 1H, Pyrid-4-*H*), 8.53 (d, *J* = 5.1 Hz, 1H, Pyrim-6-*H*), 8.48 (dt, *J* = 8.1 Hz, 1.8 Hz, 1H, Pyrid-6-*H*), 8.10 (d, *J* = 2.0, 1H, Phen-NH-6-*H*), 7.87 (dd, *J* = 4.5 Hz, 1.6 Hz, 2H, -CO-Pyrid-2,6-*H*), 7.53 (dd, *J* = 8.0 Hz, 4.2 Hz, 1H, Pyrid-5-*H*), 7.49 (dd, *J* = 8.2 Hz, 2.1 Hz, 1H, Phen-NH-4*H*), 7.45 (d, *J* = 5.2 Hz, 1H, Pyrim-5-*H*), 7.24 (d, *J* = 8.4, Phen-NH-3*H*), 2.24 (s, 3H, -C*H3*); ^{13}C-NMR (125.77 MHz, $(CD_3)_2SO$) δ = 164.26 (*C*=O), 162.08 (Pyrim-4-*C*), 161.59 (Pyrim-2-*C*), 159.97 (Pyrim-6-*C*), 151.89 (Pyrid-4-*C*), 150.73 (-CO-Pyrid-3,5-*C*), 148.68 (Pyrid-2-*C*), 142.51 (-CO-Pyrid-1-*C*), 138.38 (Phen-NH-1-*C*), 137.04 (Phen-NH-5-*C*), 134.90 (Pyrid-6-*C*), 132.64 (Pyrid-1-*C*), 130.65 (Phen-NH-3-*C*), 128.61 (Phen-NH-2-*C*), 124.28 (Pyrid-5-*C*), 122.04 (-CO-Pyrid-2,6-*C*), 117.65 (Phen-NH-6-*C*), 117.21 (Phen-NH-4-*C*), 108.08 (Pyrim-5-*C*), 18.16 (-*C*H3); R_f (PE/THF = 1/1): 0.36; $C_{22}H_{18}N_6O$; MS (ESI +): m/z = 383.0 $[M+H]^+$; colourless solid.

4-Methyl-*N*-(4-methyl-3-(4-(pyridin-3-yl)pyrimidin-2-ylamino)phenyl)benzamide (**31**)

Preparation according to general procedure (**a**) using 4-methylbenzoic acid (**21**). Yield: 30%

^{1}H-NMR (500 MHz, $(CD_3)_2SO$): δ = 10.12 (s, 1H, N*H*-CO), 9.28 (d, *J* = 2.2 Hz, 1H, Pyrid-2-*H*), 8.98 (s, 1H, Pyrim-N*H*), 8.69 (dd, *J* = 4.8 Hz, 1.6 Hz, 1H, Pyrid-4-*H*), 8.52 (d, *J* = 5.1 Hz, 1H, Pyrim-6-*H*), 8.49 (dt, *J* = 8.1 Hz, 1.8 Hz, 1H, Pyrid-6-*H*), 8.08 (d, *J* = 2.0, 1H, Phen-NH-6-*H*), 7.88 (d, *J* = 8.2 Hz, 2H, Phen-CO-2,6-*H*), 7.53 (dd, *J* = 8.0 Hz, 4.2 Hz, 1H, Pyrid-5-*H*), 7.49 (dd, *J* = 8.2 Hz, 2.1 Hz, 1H, Phen-NH-4*H*), 7.44 (d, *J* = 5.2 Hz, 1H, Pyrim-5-*H*), 7.34 (d, *J* = 8.0 Hz, 2H, Phen-CO-3,5-*H*), 7.21 (d, *J* = 8.4, Phen-NH-3*H*), 2.39 (s, 3H, -CO-Phen-C*H3*), 2.22 (s, 3H, Phen-C*H3*); ^{13}C-NMR (125.77 MHz, $(CD_3)_2SO$) δ: 165.64 (*C*=O), 162.07 (Pyrim-4-*C*), 161.66 (Pyrim-2-*C*), 159.96 (Pyrim-6-*C*), 151.87 (Pyrid-4-*C*), 148.68 (Pyrid-2-*C*), 141.90 (Phen-CO-4-*C*), 138.24 (Phen-NH-1-*C*), 137.69 (Phen-NH-5-*C*), 134.90 (Pyrid-6-*C*), 132.68 (Pyrid-1-*C*), 132.66 (Phen-CO-1-*C*), 130.48 (Phen-NH-3-*C*), 129.36 (Phen-CO-3,5-*C*), 128.12 (Phen-CO-2,6-*C*), 128.01 (Phen-NH-2-*C*), 124.26 (Pyrid-5-*C*), 117.69 (Phen-NH-6-*C*), 117.20 (Phen-NH-4-*C*), 107.97 (Pyrim-5-*C*), 21.48 (-CO-Phen-*C*H3), 18.12 (Phen-*C*H3); R_f (PE/THF = 1/1): 0.20; $C_{24}H_{21}N_5O$; MS (ESI +): m/z = 396.2 $[M+H]^+$; colourless solid.

4-Ethyl-*N*-(4-methyl-3-(4-(pyridin-3-yl)pyrimidin-2-ylamino)phenyl)benzamide (**32**)

Preparation according to general procedure (**a**) using 4-ethylbenzoic acid (**22**). Yield: 43%

^{1}H-NMR (500 MHz, $(CD_3)_2SO$): δ = 10.13 (s, 1H, N*H*-CO), 9.28 (d, *J* = 2.2 Hz, 1H, Pyrid-2-*H*), 8.98 (s, 1H, Pyrim-N*H*), 8.69 (dd, *J* = 4.8 Hz, 1.6 Hz, 1H, Pyrid-4-*H*), 8.52 (d, *J* = 5.1 Hz, 1H, Pyrim-6-*H*), 8.49 (dt, *J* = 8.1 Hz, 1.8 Hz, 1H, Pyrid-6-*H*), 8.08 (d, *J* = 2.0, 1H, Phen-NH-6-*H*), 7.89 (d, *J* = 8.3 Hz, 2H, Phen-CO-2,6-*H*), 7.53 (dd, *J* = 8.0 Hz, 4.2 Hz, 1H, Pyrid-5-*H*), 7.49 (dd, *J* = 8.2 Hz, 2.1 Hz, 1H, Phen-NH-4*H*), 7.44 (d, *J* = 5.2 Hz, 1H, Pyrim-5-*H*), 7.37 (d, *J* = 7.9 Hz, 2H, Phen-CO-3,5-*H*), 7.21 (d, *J* = 8.4, Phen-NH-3*H*), 2.69 (q, *J* = 7.6 Hz, 2H, -CO-Phen-C*H2*-CH3), 2.22 (s, 3H, Phen-C*H3*), 1.22 (t, *J* = 7.6 Hz, 3H, -CO-Phen-CH2-C*H3*); ^{13}C-NMR (125.77 MHz, $(CD_3)_2SO$) δ: 165.73 (*C*=O), 162.06 (Pyrim-4-*C*), 161.65 (Pyrim-2-*C*), 159.96 (Pyrim-6-*C*), 151.87 (Pyrid-4-*C*), 148.67 (Pyrid-2-*C*), 148.06 (Phen-CO-4-*C*), 138.24 (Phen-NH-1-*C*), 137.71 (Phen-NH-5-*C*), 134.89 (Pyrid-6-*C*), 133.00 (Phen-

CO-1-*C*), 132.68 (Pyrid-1-*C*), 130.48 (Phen-NH-3-*C*), 128.20 (Phen-CO-2,3,5,6-*C*), 128.01 (Phen-NH-2-*C*), 124.26 (Pyrid-5-*C*), 117.66 (Phen-NH-6-*C*), 117.18 (Phen-NH-4-*C*), 107.97 (Pyrim-5-*C*), 28.54 (-CO-Phen-CH_2-CH_3), 18.12 (Phen-CH_3), 15.89 (-CO-Phen-CH_2-CH_3); R_f (PE/THF = 1/1): 0.17; $C_{25}H_{23}N_5O$; MS (ESI +): m/z = 410.9 $[M+H]^+$; colourless solid.

4-*tert*-Butyl-*N*-(4-methyl-3-(4-(pyridin-3-yl)pyrimidin-2-ylamino)phenyl)-benzamide (**33**)

Preparation according to general procedure (**a**) using 4-*tert*-butylbenzoic acid (**23**). Yield: 41%

^{1}H-NMR (500 MHz, $(CD_3)_2SO$): δ = 10.13 (s, 1H, N*H*-CO), 9.28 (d, *J* = 2.2 Hz, 1H, Pyrid-2-*H*), 8.98 (s, 1H, Pyrim-N*H*), 8.69 (dd, *J* = 4.8 Hz, 1.6 Hz, 1H, Pyrid-4-*H*), 8.52 (d, *J* = 5.1 Hz, 1H, Pyrim-6-*H*), 8.49 (dt, *J* = 8.1 Hz, 1.8 Hz, 1H, Pyrid-6-*H*), 8.08 (d, *J* = 2.0, 1H, Phen-NH-6-*H*), 7.89 (d, *J* = 7.7 Hz, 2H, Phen-CO-2,6-*H*), 7.54 (d, *J* = 7.8 Hz, 2H, Phen-CO-3,5-*H*), 7.53 (dd, *J* = 8.0 Hz, 4.2 Hz, 1H, Pyrid-5-*H*), 7.49 (dd, *J* = 8.2 Hz, 2.1 Hz, 1H, Phen-NH-4*H*), 7.44 (d, *J* = 5.2 Hz, 1H, Pyrim-5-*H*), 7.21 (d, *J* = 8.4, Phen-NH-3*H*), 2.22 (s, 3H, Phen-C*H₃*), 1.32 (s, 9H, -CO-Phen-CH_2-$(CH_3)_3$); ^{13}C-NMR (125.77 MHz, $(CD_3)_2SO$) δ: 165.80 (*C*=O), 162.06 (Pyrim-4-*C*), 161.65 (Pyrim-2-*C*), 159.96 (Pyrim-6-*C*), 154.74 (Phen-CO-4-*C*), 151.87 (Pyrid-4-*C*), 148.67 (Pyrid-2-*C*), 138.25 (Phen-NH-1-*C*), 137.72 (Phen-NH-5-*C*), 134.90 (Pyrid-6-*C*), 132.88 (Phen-CO-1-*C*), 132.68 (Pyrid-1-*C*), 130.49 (Phen-NH-3-*C*), 128.03 (Phen-NH-2-*C*), 127.94 (Phen-CO-2,6-*C*), 125.61 (Phen-CO-3,5-*C*), 124.27 (Pyrid-5-*C*), 117.62 (Phen-NH-6-*C*), 117.14 (Phen-NH-4-*C*), 107.97 (Pyrim-5-*C*), 35.14 (-CO-Phen-*C*-$(CH_3)_3$), 31.42 (-CO-Phen-C-$(CH_3)_3$), 18.13 (Phen-CH_3); R_f (PE/THF = 1/1): 0.19; $C_{27}H_{27}N_5O$; MS (ESI +): m/z = 438.5 $[M+H]^+$; pale yellow solid.

N-(4-Methyl-3-(4-(pyridin-3-yl)pyrimidin-2-ylamino)phenyl)biphenyl-4-carboxamide (**34**)

Preparation according to general procedure (**a**) using 4-phenylbenzoic acid (**24**). Yield: 38%

^{1}H-NMR (500 MHz, $(CD_3)_2SO$): δ = 10.27 (s, 1H, N*H*-CO), 9.29 (d, *J* = 2.2 Hz, 1H, Pyrid-2-*H*), 9.00 (s, 1H, Pyrim-N*H*), 8.70 (dd, *J* = 4.8 Hz, 1.6 Hz, 1H, Pyrid-4-*H*), 8.53 (d, *J* = 5.1 Hz, 1H, Pyrim-6-*H*), 8.50 (dt, *J* = 8.1 Hz, 1.8 Hz, 1H, Pyrid-6-*H*), 8.12 (d, *J* = 2.0, 1H, Phen-NH-6-*H*), 8.08 (d, *J* = 8.1 Hz, 2H, -CO-Phen-2,6-*H*), 7.85 (d, *J* = 8.2 Hz, 2H, -CO-Phen-3,5-*H*), 7.78 (d, *J* = 7.7 Hz, 2H, Phen-Phen-2,6-*H*), 7.55-7.50 (m, 4H, Pyrid-5-*H*, Phen-NH-4*H*, Phen-Phen-3,5-*H*), 7.45-7.42 (m, 2H, Pyrim-5-*H*, Phen-Phen-4-*H*), 7.23 (d, *J* = 8.4, Phen-NH-3*H*), 2.24 (s, 3H, -CH_3); ^{13}C-NMR (125.77 MHz, $(CD_3)_2SO$) δ: 165.45 (*C*=O), 162.08 (Pyrim-4-*C*), 161.66 (Pyrim-2-*C*), 159.98 (Pyrim-6-*C*), 151.88 (Pyrid-4-*C*), 148.68 (Pyrid-2-*C*), 143.47 (-CO-Phen-4-*C*), 139.60 (Phen-Phen-1-*C*), 138.29 (Phen-NH-1-*C*), 137.65 (Phen-NH-5-*C*), 134.90 (Pyrid-6-*C*), 134.30 (-CO-Phen-1-*C*), 132.69 (Pyrid-1-*C*), 130.53 (Phen-NH-3-*C*), 129.54 (Phen-Phen-3,5-*C*), 128.80 (-CO-Phen-2,6-*C*), 128.61 (Phen-Phen-4-*C*), 128.14 (Phen-NH-2-*C*), 127.39 (Phen-Phen-2,6-*C*), 127.05 (-CO-Phen-3,5-*C*), 124.28 (Pyrid-5-*C*), 117.69 (Phen-NH-6-*C*), 117.21 (Phen-NH-4-*C*), 108.00 (Pyrim-5-*C*), 18.14 (-CH_3); R_f (PE/THF = 1/1): 0.13; $C_{29}H_{23}N_5O$; MS (ESI +): m/z = 458.1 $[M+H]^+$; pale yellow solid.

4-Methoxy-*N*-(4-methyl-3-(4-(pyridin-3-yl)pyrimidin-2-ylamino)phenyl)-benzamide (**35**)

Preparation according to general procedure (**a**) using 4-methoxybenzoic acid (**25**). Yield: 39%

^{1}H-NMR (500 MHz, $(CD_3)_2SO$): δ = 10.05 (s, 1H, N*H*-CO), 9.28 (d, *J* = 2.2 Hz, 1H, Pyrid-2-*H*), 8.98 (s, 1H, Pyrim-N*H*), 8.69 (dd, *J* = 4.8 Hz, 1.6 Hz, 1H, Pyrid-4-*H*), 8.52 (d, *J* = 5.1 Hz, 1H, Pyrim-6-*H*), 8.49 (dt, *J* = 8.1 Hz, 1.8 Hz, 1H, Pyrid-6-*H*), 8.07 (d, *J* = 2.0, 1H, Phen-NH-6-*H*), 7.96 (d, *J* = 8.9 Hz, 2H, Phen-CO-2,6-*H*), 7.53 (dd, *J* = 8.0 Hz, 4.2 Hz, 1H, Pyrid-5-*H*), 7.49 (dd, *J* = 8.2 Hz, 2.1 Hz, 1H, Phen-NH-4*H*), 7.44 (d, *J* = 5.2 Hz, 1H, Pyrim-5-*H*), 7.20 (d, *J* = 8.4, Phen-NH-3*H*), 7.06 (d, *J* = 8.9 Hz, 2H, Phen-CO-3,5-*H*), 3.84 (s, 3H, -O-C*H*$_3$), 2.22 (s, 3H, -C*H*$_3$); ^{13}C-NMR (125.77 MHz, $(CD_3)_2SO$) δ: 165.19 (*C*=O), 162.28 (Phen-CO-4-*C*), 162.07 (Pyrim-4-*C*), 161.67 (Pyrim-2-*C*), 159.95 (Pyrim-6-*C*), 151.87 (Pyrid-4-*C*), 148.67 (Pyrid-2-*C*), 138.22 (Phen-NH-1-*C*), 137.80 (Phen-NH-5-*C*), 134.90 (Pyrid-6-*C*), 132.68 (Pyrid-1-*C*), 130.46 (Phen-NH-3-*C*), 130.00 (Phen-CO-2,6-*C*), 127.89 (Phen-NH-2-*C*), 127.56 (Phen-CO-1-*C*), 124.26 (Pyrid-5-*C*), 117.70 (Phen-NH-6-*C*), 117.20 (Phen-NH-4-*C*), 114.04 (Phen-CO-3,5-*C*), 107.96 (Pyrim-5-*C*), 55.89 (-O-*C*H$_3$), 18.12 (-*C*H$_3$); R_f (PE/THF = 1/1): 0.17; $C_{24}H_{21}N_5O_2$; MS (ESI +): m/z = 412.0 $[M+H]^+$; pale yellow solid.

4-Chloro-*N*-(4-methyl-3-(4-(pyridin-3-yl)pyrimidin-2-ylamino)phenyl)benzamide (**36**)

Preparation according to general procedure (**a**) using 4-chlorobenzoic acid (**26**). Yield: 19%

^{1}H-NMR (500 MHz, $(CD_3)_2SO$): δ = 10.28 (s, 1H, N*H*-CO), 9.28 (d, *J* = 2.2 Hz, 1H, Pyrid-2-*H*), 8.99 (s, 1H, Pyrim-N*H*), 8.69 (dd, *J* = 4.8 Hz, 1.6 Hz, 1H, Pyrid-4-*H*), 8.52 (d, *J* = 5.1 Hz, 1H, Pyrim-6-*H*), 8.49 (dt, *J* = 8.1 Hz, 1.8 Hz, 1H, Pyrid-6-*H*), 8.08 (d, *J* = 2.0, 1H, Phen-NH-6-*H*), 7.99 (d, *J* = 8.6 Hz, 2H, Phen-CO-2,6-*H*), 7.61 (d, *J* = 8.7 Hz, 2H, Phen-CO-3,5-*H*), 7.53 (dd, *J* = 8.0 Hz, 4.2 Hz, 1H, Pyrid-5-*H*), 7.48 (dd, *J* = 8.2 Hz, 2.1 Hz, 1H, Phen-NH-4*H*), 7.44 (d, *J* = 5.2 Hz, 1H, Pyrim-5-*H*), 7.22 (d, *J* = 8.4, Phen-NH-3*H*), 2.23 (s, 3H, -C*H*$_3$); ^{13}C-NMR (125.77 MHz, $(CD_3)_2SO$) δ: 164.71 (*C*=O), 162.08 (Pyrim-4-*C*), 161.62 (Pyrim-2-*C*), 159.96 (Pyrim-6-*C*), 151.88 (Pyrid-4-*C*), 148.67 (Pyrid-2-*C*), 138.30 (Phen-NH-1-*C*), 137.41 (Phen-NH-5-*C*), 136.75 (Phen-CO-4-*C*), 134.90 (Pyrid-6-*C*), 134.22 (Phen-CO-1-*C*), 132.66 (Pyrid-1-*C*), 130.55 (Phen-NH-3-*C*), 130.06 (Phen-CO-2,6-*C*), 128.92 (Phen-CO-3,5-*C*), 128.27 (Phen-NH-2-*C*), 124.27 (Pyrid-5-*C*), 117.68 (Phen-NH-6-*C*), 117.21 (Phen-NH-4-*C*), 108.03 (Pyrim-5-*C*), 18.14 (-*C*H$_3$); R_f (PE/THF = 1/1): 0.17; $C_{23}H_{18}ClN_5O$; MS (ESI +): m/z = 416.1 $[M+H]^+$; colourless solid.[1,2]

Preparation of imatinib derivatives (mesylate, hydrate) **7-17**

General procedure (**b**) for mesylate synthesis

The respective amide **17-26** (1.0 eq.) or amine **6** (1.0 eq.) was suspended in dry methanol (50 mL/mmol **17-26**). After addition of methanesulfonic acid (2.0 eq.), the respective amide **17-26** or amine **6** dissolved yielding a yellow solution. After addition of dry diethyl ether, the respective mesylate (**28-37**) precipitated, was isolated by filtration and washed with diethyl ether. The respective mesylate (**28-37**) was dried at 80 °C and atmospheric pressure. Determination of salt composition was achieved by using NMR spectroscopy and combustion analysis.

N-(4-Methyl-3-(4-(pyridin-3-yl)pyrimidin-2-ylamino)phenyl)acetamide dimesylate-hemihydrate (**7**)

Preparation according to general procedure (**b**) using **27**. Yield: 47%

^{1}H-NMR (500 MHz, $(CD_3)_2SO$): δ = 9.88 (s, 1H, N*H*-CO), 9.35 (d, *J* = 1.8 Hz, 1H, Pyrid-2-*H*), 9.04 (s, 1H, Pyrim-N*H*), 8.83 (dd, *J* = 5.3 Hz, 1.3 Hz, 1H, Pyrid-4-*H*), 8.75 (dt, *J* = 8.1 Hz, 1.8 Hz, 1H, Pyrid-6-*H*), 8.57 (d, *J* = 6.2 Hz, 1H, Pyrim-6-*H*), 7.92 (d, *J* = 1.7, 1H, Phen-NH-6-*H*), 7.80 (dd, *J* = 8.1 Hz, 5.4 Hz, 1H, Pyrid-5-*H*), 7.50 (d, *J* = 5.2 Hz, 1H, Pyrim-5-*H*), 7.25 (dd, *J* = 8.2 Hz, 2.1 Hz, 1H, Phen-NH-4*H*), 7.15 (d, *J* = 8.4, Phen-NH-3*H*), 2.32 (s, 6H, S-CH_3), 2.19 (s, 3H, Phen-CH_3), 2.04 (s, 3H, H_3C-CO); ^{13}C-NMR (125.77 MHz, $(CD_3)_2SO$) δ: 168.59 (*C*=O), 161.57 (Pyrim-2-*C*), 160.86 (Pyrim-4-*C*), 160.26 (Pyrim-6-*C*), 149.03 (Pyrid-4-*C*), 146.07 (Pyrid-2-*C*), 138.43 (Pyrid-6-*C*), 137.98 (Phen-NH-1-*C*), 137.83 (Phen-NH-5-*C*), 134.06 (Pyrid-1-*C*), 130.76 (Phen-NH-3-*C*), 127.31 (Phen-NH-2-*C*), 125.67 (Pyrid-5-*C*), 116.40 (Phen-NH-6-*C*), 116.01 (Phen-NH-4-*C*), 108.21 (Pyrim-5-*C*), 40.22 (S-CH_3), 24.44 (H_3C-CO), 18.03 (Phen-CH_3); $C_{18}H_{17}N_5O$ x 2 CH_4O_3S x 0.5 H_2O; combustion analysis: measured (calculated): C 45.79 (46.14); H 5.00 (5.03); N 13.45 (13.45); yellow solid.

N-(4-Methyl-3-(4-(pyridin-3-yl)pyrimidin-2-ylamino)phenyl)benzamide dimesylate-hemihydrate (**8**)

Preparation according to general procedure (**b**) using **28**. Yield: 20%

^{1}H-NMR (500 MHz, $(CD_3)_2SO$): δ = 10.23 (s, 1H, N*H*-CO), 9.41 (d, *J* = 1.8 Hz, 1H, Pyrid-2-*H*), 9.12 (s, 1H, Pyrim-N*H*), 8.86 (dd, *J* = 5.3 Hz, 1.3 Hz, 1H, Pyrid-4-*H*), 8.86 (dt, *J* = 8.1 Hz, 1.8 Hz, 1H, Pyrid-6-*H*), 8.60 (d, *J* = 6.2 Hz, 1H, Pyrim-6-*H*), 8.14 (d, *J* =1.7, 1H, Phen-NH-6-*H*), 7.97 (d, *J* = 7.0 Hz, 2H, Phen-CO-2,6-*H*), 7.84 (dd, *J* = 8.1 Hz, 5.4 Hz, 1H, Pyrid-5-*H*), 7.61-7.57 (m, 1H, Phen-CO-4-*H*), 7.54 (d, *J* = 8.4 Hz, 2H, Phen-CO-3,5-*H*), 7.53 (d, *J* = 5.2 Hz, 1H, Pyrim-5-*H*), 7.47 (dd, *J* = 8.2 Hz, 2.1 Hz, 1H, Phen-NH-4*H*), 7.23 (d, *J* = 8.4, Phen-NH-3*H*), 2.33 (s, 6H, S-CH_3), 2.24 (s, 3H, -CH_3); ^{13}C-NMR (125.77 MHz, $(CD_3)_2SO$) δ: 165.90 (*C*=O), 161.44 (Pyrim-2-*C*), 160.34 (Pyrim-6-*C*), 160.23 (Pyrim-4-*C*), 148.40 (Pyrid-4-*C*), 145.53 (Pyrid-2-*C*), 139.07 (Pyrid-6-*C*), 137.99 (Phen-NH-1-*C*), 137.63 (Phen-NH-5-*C*), 135.52 (Phen-CO-1-*C*), 134.92 (Pyrid-1-*C*), 132.11 (Phen-CO-4-*C*), 130.74 (Phen-NH-3-*C*), 128.94 (Phen-CO-3,5-*C*), 128.24 (Phen-CO-2,6-*C*), 128.12 (Phen-NH-2-*C*), 125.96 (Pyrid-5-*C*), 117.78 (Phen-NH-6-*C*), 117.43 (Phen-NH-4-*C*), 108.28 (Pyrim-5-*C*), 40.19 (S-CH_3), 18.12 (-CH_3); $C_{23}H_{19}N_5O$ x 2 CH_4O_3S x 0.5 H_2O; combustion analysis: measured (calculated): C 51.77 (51.53); H 4.72 (4.84); N 12.04 (12.02); orange solid.

N-(4-Methyl-3-(4-(pyridin-3-yl)pyrimidin-2-ylamino)phenyl)-2-naphthamide dimesylate-sesquihydrate (**9**)

Preparation according to general procedure (**b**) using **29**. Yield: 25%

^{1}H-NMR (500 MHz, $(CD_3)_2SO$): δ = 10.42 (s, 1H, N*H*-CO), 9.42 (d, *J* = 1.8 Hz, 1H, Pyrid-2-*H*), 9.14 (s, 1H, Pyrim-N*H*), 8.85 (dd, *J* = 5.3 Hz, 1.3 Hz, 1H, Pyrid-4-*H*), 8.85 (dt, *J* = 8.1 Hz, 1.8 Hz, 1H, Pyrid-6-*H*), 8.61 (d, *J* = 6.2 Hz, 1H, Pyrim-6-*H*), 8.59 (s, 1H, Napht-1-*H*), 8.20 (d, *J* = 1.7, 1H, Phen-NH-6-*H*), 8.10-8.01 (m, 4H, Napht-3,4,5,8-*H*), 7.85 (dd, *J* = 8.1 Hz, 5.4 Hz, 1H, Pyrid-5-*H*), 7.67-7.61 (m, 2H, Napht-6,7-*H*), 7.54 (d, *J* = 5.2 Hz, 1H, Pyrim-5-*H*),

7.52 (dd, *J* = 8.2 Hz, 2.1 Hz, 1H, Phen-NH-4*H*), 7.25 (d, *J* = 8.4, Phen-NH-3*H*), 2.32 (s, 6H, S-C*H*$_3$), 2.25 (s, 3H, -C*H*$_3$); ^{13}C-NMR (125.77 MHz, $(CD_3)_2SO$) *δ*: 165.94 (*C*=O), 161.47 (Pyrim-2-*C*), 160.67 (Pyrim-4-*C*), 160.35 (Pyrim-6-*C*), 148.62 (Pyrid-4-*C*), 145.64 (Pyrid-2-*C*), 139.06 (Pyrid-6-*C*), 138.04 (Phen-NH-1-*C*), 137.71 (Phen-NH-5-*C*), 134.69 (Napht-4a-*C*), 134.21 (Pyrid-1-*C*), 132.84 (Napht-2-*C*), 132.55 (Napht-8a-*C*), 130.72 (Phen-NH-3-*C*), 129.56 (Napht-8-*C*), 128.55 (Napht-1-*C*), 128.46 (Napht-4-*C*), 128.36 (Napht-6-*C*), 128.26 (Napht-5-*C*), 128.15 (Phen-NH-2-*C*), 127.39 (Napht-7-*C*), 126.00 (Pyrid-5-*C*), 125.09 (Napht-3-*C*), 117.75 (Phen-NH-6-*C*), 117.46 (Phen-NH-4-*C*), 108.27 (Pyrim-5-*C*), 40.23 (S-*C*H$_3$), 18.12 (-*C*H$_3$); $C_{27}H_{21}N_5O$ x 2 CH_4O_3S x 1.5 H_2O; combustion analysis: measured (calculated): C 53.61 (53.53); H 4.56 (4.96); N 11.03 (10.76); orange solid.

N-(4-Methyl-3-(4-(pyridin-3-yl)pyrimidin-2-ylamino)phenyl)isonicotinamide dimesylate-monohydrate (**10**)

Preparation according to general procedure (**b**) using **30**. Yield: 60%

^{1}H-NMR (500 MHz, $(CD_3)_2SO$): *δ* = 10.64 (s, 1H, N*H*-CO), 9.43 (d, *J* = 1.8 Hz, 1H, Pyrid-2-*H*), 9.16 (s, 1H, Pyrim-N*H*), 8.92 (dd, *J* = 4.8 Hz, 1.5 Hz, 2H, -CO-Pyrid-3,5-*H*), 8.90 (dt, *J* = 8.1 Hz, 1.8 Hz, 1H, Pyrid-6-*H*), 8.88 (dd, *J* = 5.3 Hz, 1.3 Hz, 1H, Pyrid-4-*H*), 8.62 (d, *J* = 6.2 Hz, 1H, Pyrim-6-*H*), 8.17 (d, *J* = 1.7, 1H, Phen-NH-6-*H*), 8.11 (dd, *J* = 5.8 Hz, 1.3 Hz, 2H, -CO-Pyrid-2,6-*H*), 7.90 (dd, *J* = 8.1 Hz, 5.4 Hz, 1H, Pyrid-5-*H*), 7.56 (d, *J* = 5.2 Hz, 1H, Pyrim-5-*H*), 7.46 (dd, *J* = 8.2 Hz, 2.1 Hz, 1H, Phen-NH-4*H*), 7.27 (d, *J* = 8.4, Phen-NH-3*H*), 2.36 (s, 6H, S-C*H*$_3$), 2.25 (s, 3H, -C*H*$_3$); ^{13}C-NMR (125.77 MHz, $(CD_3)_2SO$) *δ*: 163.43 (*C*=O), 161.38 (Pyrim-2-*C*), 160.42 (Pyrim-4-*C*), 160.33 (Pyrim-6-*C*), 148.17 (-CO-Pyrid-3,5-*C*), 147.75 (Pyrid-4-*C*), 145.23 (-CO-Pyrid-1-*C*), 144.93 (Pyrid-2-*C*), 139.57 (Pyrid-6-*C*), 138.14 (Phen-NH-1-*C*), 136.86 (Phen-NH-5-*C*), 134.35 (Pyrid-1-*C*), 130.78 (Phen-NH-3-*C*), 128.77 (Phen-NH-2-*C*), 126.06 (Pyrid-5-*C*), 123.35 (-CO-Pyrid-2,6-*C*), 117.57 (Phen-NH-6-*C*), 117.31 (Phen-NH-4-*C*), 108.31 (Pyrim-5-*C*), 40.08 (S-*C*H$_3$), 18.14 (-*C*H$_3$); $C_{22}H_{18}N_6O$ x 2 CH_4O_3S x H_2O; combustion analysis: measured (calculated): C 48.31 (48.64); H 5.02 (4.76); N 14.19 (14.18); orange solid.

4-Methyl-*N*-(4-methyl-3-(4-(pyridin-3-yl)pyrimidin-2-ylamino)phenyl)benzamide dimesylate-monohydrate (**11**)

Preparation according to general procedure (**b**) using **31**. Yield: 18%

^{1}H-NMR (500 MHz, $(CD_3)_2SO$): *δ* = 10.14 (s, 1H, N*H*-CO), 9.43 (d, *J* = 1.8 Hz, 1H, Pyrid-2-*H*), 9.14 (s, 1H, Pyrim-N*H*), 8.93 (dt, *J* = 8.1 Hz, 1.8 Hz, 1H, Pyrid-6-*H*), 8.89 (dd, *J* = 5.3 Hz, 1.3 Hz, 1H, Pyrid-4-*H*), 8.61 (d, *J* = 6.2 Hz, 1H, Pyrim-6-*H*), 8.14 (d, *J* = 1.7, 1H, Phen-NH-6-*H*), 7.91 (dd, *J* = 8.1 Hz, 5.4 Hz, 1H, Pyrid-5-*H*), 7.89 (d, *J* = 8.2 Hz, 2H, Phen-CO-2,6-*H*), 7.55 (d, *J* = 5.2 Hz, 1H, Pyrim-5-*H*), 7.47 (dd, *J* = 8.2 Hz, 2.1 Hz, 1H, Phen-NH-4*H*), 7.34 (d, *J* = 8.0 Hz, 2H, Phen-CO-3,5-*H*), 7.22 (d, *J* = 8.4, Phen-NH-3*H*), 2.39 (s, 3H, -CO-Phen-C*H*$_3$), 2.34 (s, 6H, S-C*H*$_3$), 2.23 (s, 3H, Phen-C*H*$_3$); ^{13}C-NMR (125.77 MHz, $(CD_3)_2SO$) *δ*: 165.71 (*C*=O), 161.40 (Pyrim-2-*C*), 160.35 (Pyrim-6-*C*), 160.26 (Pyrim-4-*C*), 147.56 (Pyrid-4-*C*), 144.77 (Pyrid-2-*C*), 141.96 (Phen-CO-4-*C*), 139.92 (Pyrid-6-*C*), 137.89 (Phen-NH-1-*C*), 137.71 (Phen-NH-5-*C*), 134.94 (Pyrid-1-*C*), 132.61 (Phen-CO-1-*C*), 130.59 (Phen-NH-3-*C*), 129.35 (Phen-CO-3,5-*C*), 128.17 (Phen-CO-2,6-*C*), 127.94 (Phen-NH-2-*C*), 126.19 (Pyrid-5-*C*), 117.70 (Phen-NH-6-*C*), 117.40 (Phen-NH-4-*C*), 108.25 (Pyrim-5-*C*), 40.04 (S-*C*H$_3$), 21.48 (-CO-Phen-*C*H$_3$), 18.08

(Phen-CH_3); $C_{24}H_{21}N_5O$ x 2 CH_4O_3S x 1 H_2O; combustion analysis: measured (calculated): C 51.46 (51.56); H 4.90 (5.16); N 11.40 (11.56); orange solid.

4-Ethyl-*N*-(4-methyl-3-(4-(pyridin-3-yl)pyrimidin-2-ylamino)phenyl)benzamide dimesylate-monohydrate (**12**)

Preparation according to general procedure (**b**) using **32**. Yield: 48%

^{1}H-NMR (500 MHz, $(CD_3)_2SO$): δ = 10.15 (s, 1H, N*H*-CO), 9.45 (d, *J* = 1.8 Hz, 1H, Pyrid-2-*H*), 9.17 (s, 1H, Pyrim-N*H*), 8.99 (dt, *J* = 8.1 Hz, 1.8 Hz, 1H, Pyrid-6-*H*), 8.92 (dd, *J* = 5.3 Hz, 1.3 Hz, 1H, Pyrid-4-*H*), 8.63 (d, *J* = 6.2 Hz, 1H, Pyrim-6-*H*), 8.15 (d, *J* = 1.7, 1H, Phen-NH-6-*H*), 7.96 (dd, *J* = 8.0 Hz, 4.2 Hz, 1H, Pyrid-5-*H*), 7.90 (d, *J* = 8.3 Hz, 2H, Phen-CO-2,6-*H*), 7.56 (d, *J* = 5.2 Hz, 1H, Pyrim-5-*H*), 7.45 (dd, *J* = 8.2 Hz, 2.1 Hz, 1H, Phen-NH-4*H*), 7.37 (d, *J* = 7.9 Hz, 2H, Phen-CO-3,5-*H*), 7.22 (d, *J* = 8.4, Phen-NH-3*H*), 2.69 (q, *J* = 7.6 Hz, 2H, -CO-Phen-CH_2-CH_3), 2.35 (s, 6H, S-CH_3), 2.23 (s, 3H, Phen-CH_3), 1.22 (t, *J* = 7.6 Hz, 3H, -CO-Phen-CH_2-CH_3); ^{13}C-NMR (125.77 MHz, $(CD_3)_2SO$) δ: 165.80 (*C*=O), 161.35 (Pyrim-2-*C*), 160.39 (Pyrim-6-*C*), 160.14 (Pyrim-4-*C*), 148.12 (Phen-CO-4-*C*), 147.02 (Pyrid-4-*C*), 144.28 (Pyrid-2-*C*), 140.64 (Pyrid-6-*C*), 137.85 (Phen-NH-1-*C*), 137.73 (Phen-NH-5-*C*), 132.94 (Phen-CO-1-*C*, Pyrid-1-*C*), 130.68 (Phen-NH-3-*C*), 128.32 (Phen-CO-2,6-*C*), 128.27 (Phen-CO-3,5-*C*), 127.92 (Phen-NH-2-*C*), 126.50 (Pyrid-5-*C*), 117.72 (Phen-NH-6-*C*), 117.44 (Phen-NH-4-*C*), 108.31 (Pyrim-5-*C*), 40.16 (S-CH_3), 28.60 (-CO-Phen-CH_2-CH_3), 17.98 (Phen-CH_3), 15.87 (-CO-Phen-CH_2-CH_3); $C_{25}H_{23}N_5O$ x 2 CH_4O_3S x 1 H_2O; combustion analysis: measured (calculated): C 52.29 (52.33); H 5.05 (5.37); N 11.55 (11.30); orange solid.

4-*tert*-Butyl-*N*-(4-methyl-3-(4-(pyridin-3-yl)pyrimidin-2-ylamino)phenyl)-benzamide dimesylate-hemihydrate (**13**)

Preparation according to general procedure (**b**) using **33**. Yield: 56%

^{1}H-NMR (500 MHz, $(CD_3)_2SO$): δ = 10.16 (s, 1H, N*H*-CO), 9.46 (d, *J* = 1.8 Hz, 1H, Pyrid-2-*H*), 9.18 (s, 1H, Pyrim-N*H*), 9.00 (dt, *J* = 8.1 Hz, 1.8 Hz, 1H, Pyrid-6-*H*), 8.92 (dd, *J* = 5.3 Hz, 1.3 Hz, 1H, Pyrid-4-*H*), 8.63 (d, *J* = 6.2 Hz, 1H, Pyrim-6-*H*), 8.15 (d, *J* = 1.7, 1H, Phen-NH-6-*H*), 7.98 (dd, *J* = 8.1 Hz, 5.4 Hz, 1H, Pyrid-5-*H*), 7.90 (d, *J* = 7.7 Hz, 2H, Phen-CO-2,6-*H*), 7.57 (d, *J* = 5.2 Hz, 1H, Pyrim-5-*H*), 7.55 (d, *J* = 7.8 Hz, 2H, Phen-CO-3,5-*H*), 7.46 (dd, *J* = 8.2 Hz, 2.1 Hz, 1H, Phen-NH-4*H*), 7.22 (d, *J* = 8.4, Phen-NH-3*H*), 2.35 (s, 6H, S-CH_3), 2.23 (s, 3H, Phen-CH_3), 1.33 (s, 9H, -CO-Phen-CH_2-(CH_3)$_3$); ^{13}C-NMR (125.77 MHz, $(CD_3)_2SO$) δ: 165.87 (*C*=O), 161.33 (Pyrim-2-*C*), 160.42 (Pyrim-6-*C*), 160.04 (Pyrim-4-*C*), 154.80 (Phen-CO-4-*C*), 146.86 (Pyrid-4-*C*), 144.14 (Pyrid-2-*C*), 140.89 (Pyrid-6-*C*), 137.84 (Phen-NH-1-*C*), 137.74 (Phen-NH-5-*C*), 134.80 (Pyrid-1-*C*), 132.81 (Phen-CO-1-*C*), 130.71 (Phen-NH-3-*C*), 127.98 (Phen-CO-2,6-*C*), 127.91 (Phen-NH-2-*C*), 126.62 (Pyrid-5-*C*), 125.69 (Phen-CO-3,5-*C*), 117.66 (Phen-NH-6-*C*), 117.42 (Phen-NH-4-*C*), 108.33 (Pyrim-5-*C*), 40.18 (S-CH_3), 35.15 (-CO-Phen-*C*-(CH_3)$_3$), 31.42 (-CO-Phen-C-(CH_3)$_3$), 18.08 (Phen-CH_3); $C_{27}H_{27}N_5O$ x 2 CH_4O_3S x 0.5 H_2O; combustion analysis: measured (calculated): C 54.91 (54.53); H 5.60 (5.68); N 10.71 (10.96); orange solid.

N-(4-Methyl-3-(4-(pyridin-3-yl)pyrimidin-2-ylamino)phenyl)biphenyl-4-carboxamide dimesylate-monohydrate (**14**)

Preparation according to general procedure (**b**) using **34**. Yield: 44%

^{1}H-NMR (500 MHz, $(CD_3)_2SO$): δ = 10.28 (s, 1H, N*H*-CO), 9.41 (d, *J* = 1.8 Hz, 1H, Pyrid-2-*H*), 9.12 (s, 1H, Pyrim-N*H*), 8.85 (dt, *J* = 8.1 Hz, 1.8 Hz, 1H, Pyrid-6-*H*), 8.84 (dd, *J* = 5.3 Hz, 1.3 Hz, 1H, Pyrid-4-*H*), 8.59 (d, *J* = 6.2 Hz, 1H, Pyrim-6-*H*), 8.17 (d, *J* = 1.7, 1H, Phen-NH-6-*H*), 8.08 (d, *J* = 8.1 Hz, 2H, -CO-Phen-2,6-*H*), 7.85 (d, *J* = 8.2 Hz, 2H, -CO-Phen-3,5-*H*), 7.83 (dd, *J* = 8.1 Hz, 5.4 Hz, 1H, Pyrid-5-*H*), 7.77 (d, *J* = 7.7 Hz, 2H, Phen-Phen-2,6-*H*), 7.53 (d, *J* = 5.2 Hz, 1H, Pyrim-5-*H*), 7.51 (d, *J* = 8.7 Hz, 2H, Phen-Phen-3,5-*H*), 7.50 (dd, *J* = 8.2 Hz, 2.1 Hz, 1H, Phen-NH-4*H*), 7.45-7.43 (m, 1H, Phen-Phen-4-*H*), 7.24 (d, *J* = 8.4, Phen-NH-3*H*), 2.32 (s, 6H, S-C*H₃*), 2.24 (s, 3H, -C*H₃*); ^{13}C-NMR (125.77 MHz, $(CD_3)_2SO$) δ: 165.50 (*C*=O), 161.47 (Pyrim-2-*C*), 160.37 (Pyrim-6-*C*), 160.22 (Pyrim-4-*C*), 148.63 (Pyrid-4-*C*), 145.84 (Pyrid-2-*C*), 143.50 (-CO-Phen-4-*C*), 139.59 (Phen-Phen-1-*C*), 138.81 (Pyrid-6-*C*), 138.02 (Phen-NH-1-*C*), 137.66 (Phen-NH-5-*C*), 134.26 (-CO-Phen-1-*C*), 134.10 (Pyrid-1-*C*), 130.79 (Phen-NH-3-*C*), 129.70 (Phen-Phen-3,5-*C*), 128.95 (-CO-Phen-2,6-*C*), 128.71 (Phen-Phen-4-*C*), 128.07 (Phen-NH-2-*C*), 127.51 (Phen-Phen-2,6-*C*), 127.15 (-CO-Phen-3,5-*C*), 125.95 (Pyrid-5-*C*), 117.79 (Phen-NH-6-*C*), 117.53 (Phen-NH-4-*C*), 108.30 (Pyrim-5-*C*), 40.23 (S-*C*H₃), 18.11 (-CH₃); $C_{29}H_{23}N_5O$ x 2 CH_4O_3S x 1 H_2O; combustion analysis: measured (calculated): C 56.01 (55.76); H 5.18 (4.98); N 10.58 (10.49); orange solid.

4-Methoxy-*N*-(4-methyl-3-(4-(pyridin-3-yl)pyrimidin-2-ylamino)phenyl)-benzamide monomesylate (**15**)

Preparation according to general procedure (**b**) using **35**. Yield: 65%

^{1}H-NMR (500 MHz, $(CD_3)_2SO$): δ = 10.06 (s, 1H, N*H*-CO), 9.39 (d, *J* = 1.8 Hz, 1H, Pyrid-2-*H*), 9.09 (s, 1H, Pyrim-N*H*), 8.84 (dd, *J* = 5.3 Hz, 1.3 Hz, 1H, Pyrid-4-*H*), 8.81 (dt, *J* = 8.1 Hz, 1.8 Hz, 1H, Pyrid-6-*H*), 8.59 (d, *J* = 6.2 Hz, 1H, Pyrim-6-*H*), 8.11 (d, *J* = 1.7, 1H, Phen-NH-6-*H*), 7.97 (d, *J* = 8.9 Hz, 2H, Phen-CO-2,6-*H*), 7.80 (dd, *J* = 8.1 Hz, 5.4 Hz, 1H, Pyrid-5-*H*), 7.52 (d, *J* = 5.2 Hz, 1H, Pyrim-5-*H*), 7.45 (dd, *J* = 8.2 Hz, 2.1 Hz, 1H, Phen-NH-4*H*), 7.21 (d, *J* = 8.4, Phen-NH-3*H*), 7.06 (d, *J* = 8.9 Hz, 2H, Phen-CO-3,5-*H*), 3.84 (s, 3H, -O-C*H₃*), 2.32 (s, 3H, S-C*H₃*), 2.23 (s, 3H, -C*H₃*); ^{13}C-NMR (125.77 MHz, $(CD_3)_2SO$) δ: 165.24 (*C*=O), 162.30 (Phen-CO-4-*C*), 161.48 (Pyrim-2-*C*), 160.81 (Pyrim-4-*C*), 160.18 (Pyrim-6-*C*), 148.70 (Pyrid-4-*C*), 145.86 (Pyrid-2-*C*), 138.53 (Pyrid-6-*C*), 137.97 (Phen-NH-1-*C*), 137.80 (Phen-NH-5-*C*), 133.99 (Pyrid-1-*C*), 130.53 (Phen-NH-3-*C*), 130.04 (Phen-CO-2,6-*C*), 127.83 (Phen-NH-2-*C*), 127.52 (Phen-CO-1-*C*), 125.66 (Pyrid-5-*C*), 117.70 (Phen-NH-6-*C*), 117.34 (Phen-NH-4-*C*), 114.04 (Phen-CO-3,5-*C*), 108.16 (Pyrim-5-*C*), 55.90 (-O-CH₃), 40.29 (S-*C*H₃), 18.09 (-*C*H₃); $C_{24}H_{21}N_5O_2$ x CH_4O_3S; combustion analysis: measured (calculated): C 58.91 (59.16); H 4.60 (4.96); N 14.16 (13.80); yellow solid.

4-Chloro-*N*-(4-methyl-3-(4-(pyridin-3-yl)pyrimidin-2-ylamino)phenyl)benzamide dimesylate-sesquihydrate (**16**)

Preparation according to general procedure (**b**) using **36**. Yield: 43%

^{1}H-NMR (500 MHz, $(CD_3)_2SO$): δ = 10.29 (s, 1H, N*H*-CO), 9.38 (d, *J* = 1.8 Hz, 1H, Pyrid-2-*H*), 9.09 (s, 1H, Pyrim-N*H*), 8.82 (dd, *J* = 5.3 Hz, 1.3 Hz, 1H, Pyrid-4-*H*), 8.77 (dt, *J* = 8.1 Hz, 1.8 Hz, 1H, Pyrid-6-*H*), 8.58 (d, *J* = 6.2 Hz,

1H, Pyrim-6-*H*), 8.12 (d, *J* = 1.7, 1H, Phen-NH-6-*H*), 8.00 (d, *J* = 8.6 Hz, 2H, Phen-CO-2,6-*H*), 7.78 (dd, *J* = 8.0 Hz, 4.2 Hz, 1H, Pyrid-5-*H*), 7.62 (d, *J* = 8.7 Hz, 2H, Phen-CO-3,5-*H*), 7.51 (d, *J* = 5.2 Hz, 1H, Pyrim-5-*H*), 7.46 (dd, *J* = 8.2 Hz, 2.1 Hz, 1H, Phen-NH-4*H*), 7.23 (d, *J* = 8.4, Phen-NH-3*H*), 2.32 (s, 6H, S-CH_3), 2.24 (s, 3H, -CH_3); ^{13}C-NMR (125.77 MHz, $(CD_3)_2SO$) δ: 164.75 (*C*=O), 161.47 (Pyrim-2-*C*), 160.28 (Pyrim-6-*C*), 160.17 (Pyrim-4-*C*), 149.15 (Pyrid-4-*C*), 146.22 (Pyrid-2-*C*), 138.20 (Pyrid-6-*C*), 137.70 (Phen-NH-1-*C*), 137.39 (Phen-NH-5-*C*), 136.78 (Phen-CO-4-*C*), 134.18 (Phen-CO-1-*C*), 133.86 (Pyrid-1-*C*), 130.73 (Phen-NH-3-*C*), 130.20 (Phen-CO-2,6-*C*), 129.02 (Phen-CO-3,5-*C*), 128.25 (Phen-NH-2-*C*), 125.59 (Pyrid-5-*C*), 117.78 (Phen-NH-6-*C*), 117.42 (Phen-NH-4-*C*), 108.28 (Pyrim-5-*C*), 40.24 (S-*C*H$_3$), 18.10 (-*C*H$_3$); $C_{23}H_{18}ClN_5O$ x 2 CH_4O_3S x 1.5 H_2O; combustion analysis: measured (calculated): C 47.36 (47.28); H 4.40 (4.60); N 11.28 (11.03); orange solid.

6-methyl-*N*^{1}-(4-(pyridin-3-yl)pyrimidin-2-yl)benzene-1,3-diamine mono-mesylate-hemihydrate (**17**)

Preparation according to general procedure (**b**) using **6**. Yield: 24%

^{1}H-NMR (500 MHz, $(CD_3)_2SO$): δ = 9.72 (s, br, 3H, -NH_3), 9.31 (d, *J* = 2.2 Hz, 1H, Pyrid-2-*H*), 9.10 (s, 1H, Pyrim-N*H*), 8.76 (dd, *J* = 4.8 Hz, 1.6 Hz, 1H, Pyrid-4-*H*), 8.58 (d, *J* = 5.1 Hz, 1H, Pyrim-6-*H*), 8.51 (dt, *J* = 8.1 Hz, 1.8 Hz, 1H, Pyrid-6-*H*), 7.69 (d, *J* = 1.7, 1H, Phen-NH-6-*H*), 7.61 (dd, *J* = 8.0 Hz, 4.2 Hz, 1H, Pyrid-5-*H*), 7.53 (d, *J* = 5.2 Hz, 1H, Pyrim-5-*H*), 7.35 (d, *J* = 8.2, Phen-NH-3*H*), 7.02 (dd, *J* = 8.0 Hz, 2.1 Hz, 1H, Phen-NH-4*H*), 2.31 (s, 3H, S-CH_3), 2.29 (s, 3H, -CH_3); ^{13}C-NMR (125.77 MHz, $(CD_3)_2SO$) δ: 162.04 (Pyrim-4-*C*), 161.13 (Pyrim-2-*C*), 160.05 (Pyrim-6-*C*), 151.51 (Pyrid-4-*C*), 148.22 (Pyrid-2-*C*), 139.31 (Phen-NH-1-*C*), 135.53 (Pyrid-6-*C*), 132.69 (Pyrid-1-*C*), 131.83 (Phen-NH-3-*C*), 131.20 (Phen-NH-2-*C*), 124.60 (Pyrid-5-*C*), 118.45 (Phen-NH-4-*C*, Phen-NH-6-*C*), 108.87 (Pyrim-5-*C*), 40.24 (S-*C*H$_3$), 18.21 (-*C*H$_3$); $C_{16}H_{15}N_5$ x CH_4O_3S x 0.5 H_2O; combustion analysis: measured (calculated): C 53.17 (53.39); H 5.16 (5.27); N 18.23 (18.31); pale yellow solid.

***In vitro* biological evaluation**

TGR5 assay

Cellular TGR5 modulation of compounds **4** and **16** (1 and 10 µM each) was conducted by Eurofins (Brussels, Belgium) in two independent repeats.

WST-1 assay

WST-1 assay (Roche Diagnostics International AG, Rotkreuz, Schweiz) was performed according to manufacturer's protocol and as described previously. In brief, HepG2 cells were seeded in DMEM high glucose, supplemented with SP (1 mM), penicillin (100 U/mL), streptomycin (100 µg/mL) and 10% FCS in 96-well plates ($3{\cdot}10^4$ cells/well). After 24 h, medium was changed to DMEM high glucose, supplemented with penicillin (100 U/mL), streptomycin (100 µg/mL) and 1% charcoal stripped FCS and cells were incubated with **4** or **16** (final concentrations 0.1 µM, 1 µM, 10 µM and 50 µM), **1** (50 µM), **1** (50 µM) + **4** (10 µM) or **1** (50 µM) + **16** (0.3 µM) and DMEM/1% DMSO as negative controls. After 48 h (or 24 h), WST reagent (Roche Diagnostics International AG) was added to each well according to manufacturer's instructions. After 45 min incubation, absorption (450 nm/ reference: 620 nm) was determined with a Tecan Infinite M200 luminometer (Tecan Deutschland GmbH). Each experiment was repeated at least three times in triplicates. Results (expressed as mean percent of untreated control ± SEM; n ≥ 3; DMSO (0.1%) = 100%) **4**: 0.1 µM: 98 ± 9%, 1 µM: 80 ± 9%, 10 µM: 78 ± 3%, 50 µM: 65 ± 1%. **16**: 0.1 µM: 113 ± 1%, 0.3 µM: 107 ± 7%, 1 µM: 112 ± 3%, 10 µM: 92 ± 2%, 50 µM: 87 ± 2%; **1** (50 µM): 88 ± 5%; **1** (50 µM) + **4** (10 µM): 77 ± 7%; **1** (50 µM) + **16** (0.3 µM): 93 ± 7%.

Metabolism Assay

The solubilized test compound **4** or **16** (5 µL, final concentration 10 µM in phosphate buffer (0.1 M, pH 7.4)) was preincubated at 37 °C in 432 µL of phosphate buffer (0.1 M, pH 7.4) together with a 50 µL NADPH regenerating system (30 mM glucose-6-phosphate, 4 U/mL glucose-6-phosphate dehydrogenase, 10 mM NADP, 30 mM $MgCl_2$). After 5 min, the reaction was started by the addition of 13 µL of microsome mix from the liver of Sprague−Dawley rats (Invitrogen; 20 mg protein/mL in 0.1 M phosphate buffer) in a shaking water bath at 37 °C. The reaction was stopped by addition of 250 µL of ice-cold methanol at 0, 15, 30 and 60 min. The samples were diluted with 250 µL of DMSO and centrifuged at 10000 g for 5 min at 4 °C. The supernatants were analyzed and test compound was quantified by HPLC: mobile phase: MeOH 83%/H_2O 17%/formic acid 0.1%; flow-rate: 1 mL/min; stationary phase: MultoHigh Phenyl phase, 5 µm, 250 × 4, precolumn, phenyl, 5 µm, 20 × 4; detection wavelength: 330 and 254 nm; injection volume: 50 µL. Control samples were performed to check the stability of **4** or **16** in the reaction mixture: first control was without NADPH, which is needed for the enzymatic activity of the microsomes, second control was with inactivated microsomes (incubated for 20 min at 90 °C), third control was without test compound **4** or **16** (to determine the baseline). The amounts of the test compound **4** or **16** were quantified by an external calibration curve, where data are expressed as means ± SEM of single determinations

obtained in three independent experiments. The metabolism experiment showed the following results (expressed as mean percent of remaining compound ± SEM; n=3): **4**: 0 min: 100 ± 0%, 15 min: 86 ± 3%, 30 min: 74 ± 1%, 60 min: 61 ± 3%. **16**: 0 min: 100 ± 0%, 15 min: 92 ± 1%, 30 min: 88 ± 1%, 60 min: 86 ± 1%.
